# Multiparametric Analysis of Cell Shape Demonstrates that β-PIX Directly Couples YAP Activation to Extracellular Matrix Adhesion

**DOI:** 10.1016/j.cels.2016.11.015

**Published:** 2017-01-25

**Authors:** Julia E. Sero, Chris Bakal

**Affiliations:** 1 Chester Beatty Laboratories, Division of Cancer Biology, Institute of Cancer Research, 237 Fulham Road, London SW3 6JB, UK

**Keywords:** YAP, beta-PIX, Rac1, Cdc42, FAK, adhesion, cell shape, mechanobiology, morphology, breast cancer

## Abstract

Mechanical signals from the extracellular matrix (ECM) and cellular geometry regulate the nuclear translocation of transcriptional regulators such as Yes-associated protein (YAP). Elucidating how physical signals control the activity of mechanosensitive proteins poses a technical challenge, because perturbations that affect cell shape may also affect protein localization indirectly. Here, we present an approach that mitigates confounding effects of cell-shape changes, allowing us to identify direct regulators of YAP localization. This method uses single-cell image analysis and statistical models that exploit the naturally occurring heterogeneity of cellular populations. Through systematic depletion of all human kinases, Rho family GTPases, GEFs, and GTPase activating proteins (GAPs), together with targeted chemical perturbations, we found that β-PIX, a Rac1/Ccd42 GEF, and PAK2, a Rac1/Cdc42 effector, drive both YAP activation and cell-ECM adhesion turnover during cell spreading. Our observations suggest that coupling YAP to adhesion dynamics acts as a mechano-timer, allowing cells to rapidly tune gene expression in response to physical signals.

## Highlights

• Multiparametric analysis of cell morphology identifies direct regulators of YAP
• Rac1/Cdc42 signaling through β-PIX couples YAP to cell-matrix adhesion
• YAP is regulated through FAK-dependent and -independent mechanisms during spreading
• YAP is uncoupled from adhesion dynamics in triple-negative breast cancer cells

## Graphical Abstract

**Figure:**
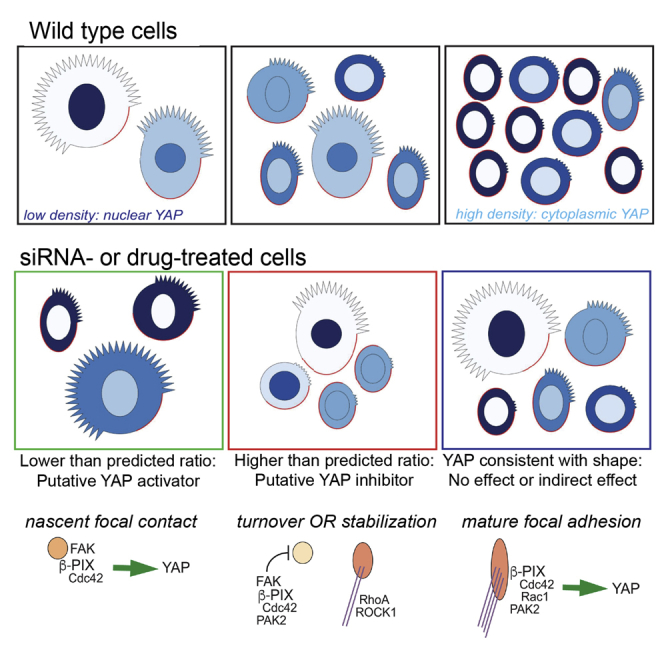

## Teaser

A statistical model is used to control for the effects of cell shape on protein localization in an RNAi screen, revealing regulators of the transcriptional coactivator Yes-associated protein (YAP). This work illustrates how the naturally occurring heterogeneity of cellular populations can be harnessed to distinguish indirect from direct effects.

## Introduction

Many fundamental cellular processes, such as proliferation and motility, are sensitive to cell shape and mechanical forces. Long-range mechanical signals transmitted through the cytoskeleton via cell-cell and cell-extracellular matrix (ECM) adhesions, as well as smaller-scale cellular distortions, can give rise to changes in gene expression (Charras and Sahai, 2014, Chen et al., 1997, Kilian et al., 2010, Watt et al., 1988). Elucidating the pathways that link cell shape and gene expression is challenging, because perturbations that are used to establish causal relationships, such as gene depletion, often lead to changes in cell morphology. These changes may have independent, and therefore indirect, effects on transcriptional programs. Such indirect effects may be a source of false positives and negatives in genetic screens and even account for irreproducibility that has been attributed to off-target effects (Barr and Bakal, 2012, Snijder et al., 2012).

Physical cues can be transduced into changes in gene expression by controlling the localization of transcription factors. YAP (Yes-associated protein) is a transcriptional coactivator that was first identified as a regulator of organ size in *Drosophila* (Meng et al., 2016). YAP, and its homolog, TAZ/WWTR1, have gained prominence in recent years as mechanosensors that drive mammalian cell growth, proliferation, differentiation, and tumorigenesis (Piccolo et al., 2014). When phosphorylated, YAP is sequestered in the cytoplasm through binding to 14-3-3 proteins and angiomotin (Kanai et al., 2000, Mana-Capelli et al., 2014). Cell distortion and mechanical forces, in addition to chemical stimuli, can trigger dephosphorylation of YAP, which allows it to enter the nucleus, bind transcription factors, and modulate gene expression (Dupont et al., 2011, Galli et al., 2015, Sansores-Garcia et al., 2011, Wada et al., 2011, Zhao et al., 2012). YAP is best known to be regulated by LATS1/2-mediated phosphorylation downstream of the Hippo pathway (Meng et al., 2016), but it is also subject to large tumor suppressor kinase (LATS)-independent regulation, e.g., via RhoA and F-actin (Halder et al., 2012). Understanding how these pathways converge to regulate YAP activity will give insight into how cells integrate diverse, and sometimes contradictory, signals to give rise to complex behaviors.

We previously used Bayesian inference models to quantify relationships between cell shape and transcription factor localization (Sero et al., 2015). Here, we used image-based analysis and multivariate regression models that exploit the naturally occurring variability present in wild-type cells to model the relationship between YAP localization and cell shape in order to identify proteins that directly regulate YAP. We found that YAP nuclear localization appears to be coupled to the generation of dynamic focal contacts and focal adhesions through the Rac1/Cdc42 guanine nucleotide exchange factor (GEF) β-PIX in non-tumor cells. Because β-PIX and PAK2 also regulate adhesion turnover, and thus the termination of signaling downstream of focal adhesions (Feng et al., 2004, Kuo et al., 2011, Zhao et al., 2000), this GTPase signaling axis may function as a “mechano-timer” whereby YAP activation is tightly coupled to physical signals and constrained by focal adhesion dynamics.

## Results

### Image-Based RNAi Screen and Normalization of Density-Sensitive Features

To identify proteins that couple YAP dynamics to cell shape, we analyzed YAP localization and morphology in MCF10A mammary epithelial cells following systematic depletion of all Rho family GTPases, GEFs, GTPase activating proteins (GAPs), and the entire kinome (950 gene targets) using pooled small interfering RNA (siRNA) (Dharmacon siGenome; siG). Cells were reverse transfected in 384-well plates, fixed after 72 hr, and stained for DNA, F-actin, and YAP. The antibody used in these studies (Santa Cruz Biotechnology, 63.7) can bind both YAP and TAZ, but the majority of the fluorescent signal came from YAP (Figure S1). Automated image analysis was used to segment cells and extract over 100 shape, context, and regional intensity features (see STAR Methods).

**Figure 1:**
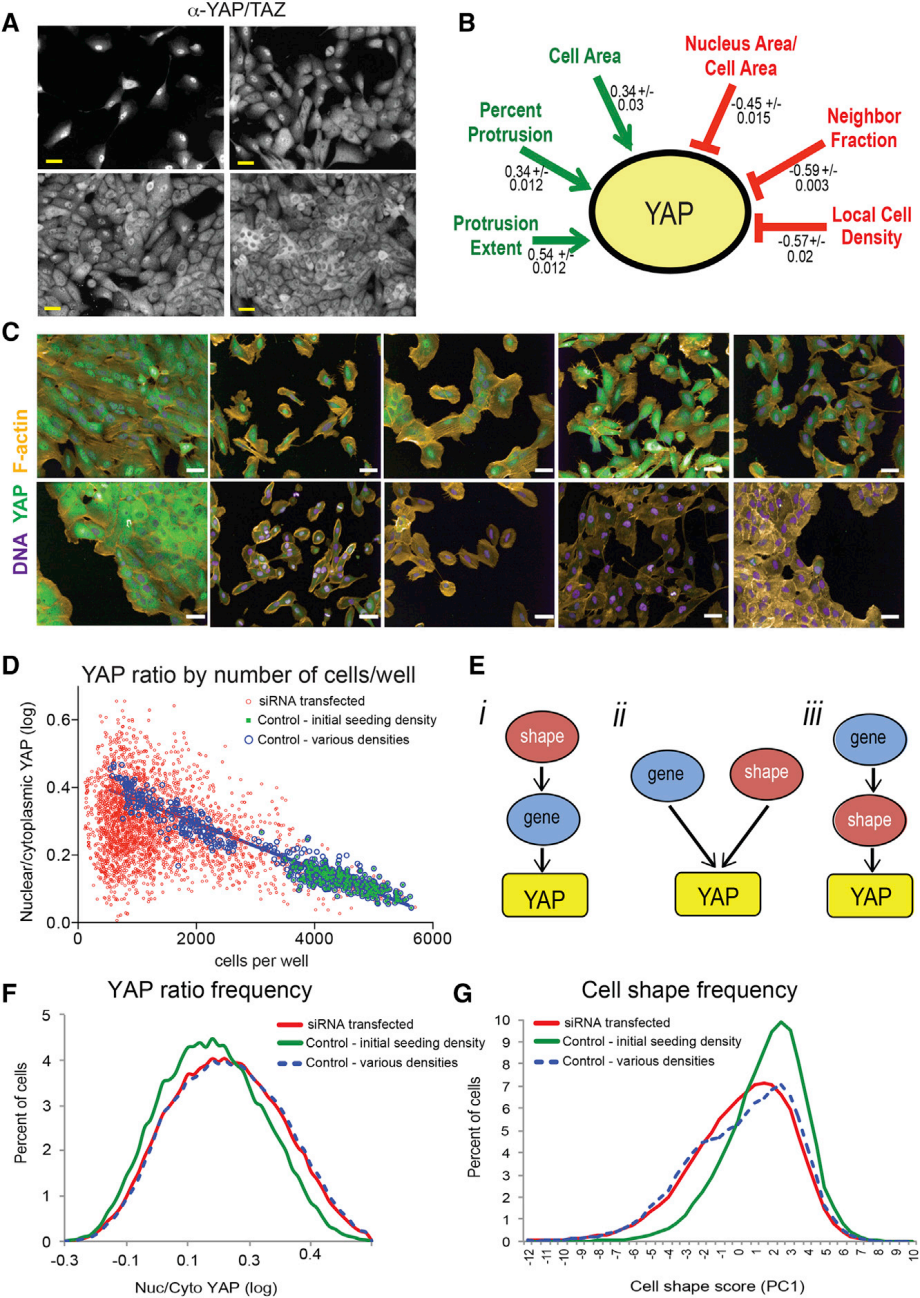
Strategy for Identifying Perturbations that Specifically Affect YAP Localization (A) MCF10A cells at low to high densities labeled with anti-YAP antibody. Scale bar, 20 μm. (B) YAP ratios are positively and negatively correlated with cell morphology features. Pearson’s correlation coefficients for ten sets of wild-type cells seeded at low to high density ± SD are indicated (n = 24,000 to ≥100,000 cells per set). (C) Diverse morphologies in siRNA-transfected cells. Scale bar, 20 μm. (D) Average YAP ratios were negatively correlated with cell number in wild-type cells seeded at different densities but varied widely in siRNA-transfected cells. (E) Perturbations where YAP ratios were not consistent with cell shape suggest that the target may “directly” couple YAP to cell shape (i) or regulate YAP independently of shape (ii). Changes in YAP localization that could be explained by changes in morphology suggest “indirect” effects (iii). (F) Frequencies of the nuclear/cytoplasmic YAP ratio in single cells. (G) Frequencies of cell shape in single cells, as shown by the first principal component (PC1) score of morphology features.

The proportion of YAP in the nucleus (log_10_ of mean nuclear intensity/mean perinuclear intensity), referred to here as the “YAP ratio,” decreased with cell density in wild-type MCF10As (Figure 1A). In single cells, YAP ratio was positively correlated with cell area and measures of protrusiveness (percent protrusion and protrusion extent [ProX]) and negatively correlated with cell-cell contact (neighbor fraction [NF]), crowding (local cell density [LCD]), and the nuclear area/cell area ratio (A_nuc_/A_cell_) (n > 20,000 cells) (Figure 1B). Many siRNAs affected cell-shape features (Figure 1C), and the majority of siRNA-transfected wells had fewer cells than mock-transfected controls (Figure 1D).

The differences in cell shape and density meant that we could not identify hits by simply comparing YAP ratios in siRNA- and mock-transfected wells. To identify genes that directly regulate YAP and/or couple its localization to morphological cues (Figure 1E, i and ii) we wanted to filter out cases where changes in YAP localization were consistent with changes in density and shape (Figure 1 E, iii). We used a two-step method to identify hits. First, we generated statistical models to describe the relationship between the YAP ratio and multiple shape features in wild-type cells. Mock-transfected control wells were seeded at a 4-fold range of densities so that wild-type populations spanned the range of YAP ratios (Figure 1F) and shape phenotypes (Figure 1G) observed in screen. Statistical models could therefore be generated entirely from cells with ostensibly functional YAP regulatory machinery. Second, we applied these models to all siRNA-treated cells and looked for wells in which YAP localization was not accurately predicted by cell shape. The observation that RNAi enriched for morphological phenotypes already present in wild-type cells, rather than generating novel phenotypes, is consistent with our previous studies (Sailem et al., 2014, Yin et al., 2013, Yin et al., 2014).

**Figure 2:**
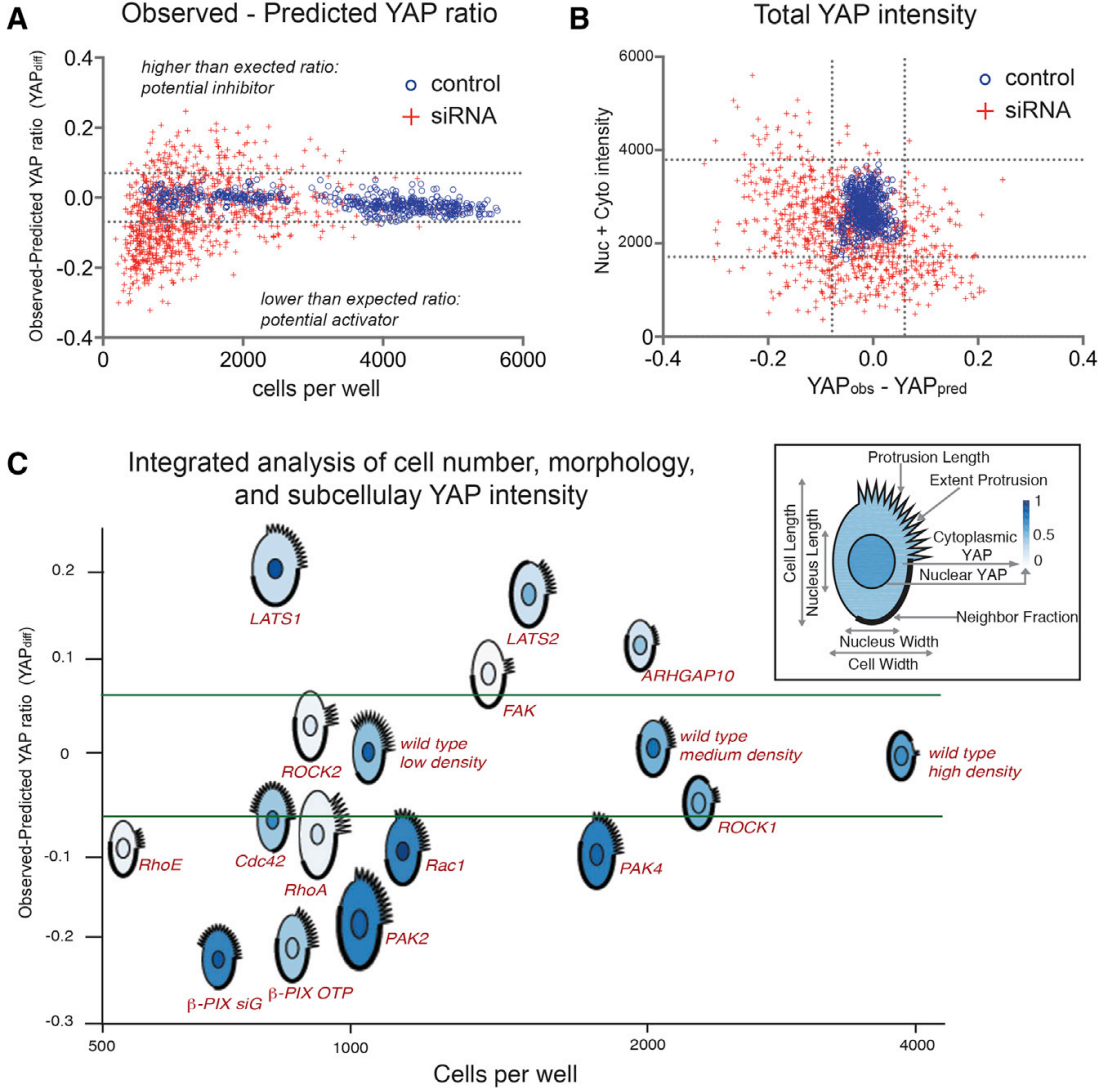
Analysis of RNAi Screen for Perturbations in the Relationship between YAP Localization and Cell Shape (A) Difference between observed YAP ratios and YAP ratios predicted from morphological features (YAP_diff_). (B) Total YAP intensities as a function of YAP_diff_. Blue circles: average of each control well (n = 554 wells, n = 273-3382 cells/well). Red crosses: average of replicate siRNA-transfected wells. Dashed lines: 2 SDs of control wells. (C) Glyphs represent average normalized shape features and subcellular YAP intensities (see inset). Relative positions correspond to average YAP_diff_ (y axis) and numbers of cells per well (x axis).

Multiparametric linear regression (MLR) models incorporating the six most highly correlated shape features were derived from wild-type cells in each plate (n = 25,000–100,000) (Figure 1B; STAR Methods). MLR equations were applied to each cell in the screen to calculate the predicted YAP ratio (YAP_pred_) and the difference between the observed and predicted YAP ratios (YAP_diff_). Average YAP_diff_ values were then used to score hits: targets with high YAP_diff_ scores as potential inhibitors and targets with low YAP_diff_ as potential activators. Figure 2A shows YAP_diff_ for knockdown and control wells plotted by the number of cells per well. The hit threshold was set as ±2 SD of control wells (YAP_diff_ ± 0.06) for two out of two (kinases) or three out of four (GGG) replicate wells. 76% of gene targets scored as hits in two independent screens (Data S1; Figure S3). Density-sensitive shape features, such as cell area and NF, were also normalized to “standard curves” using control wells (Data S2; Figure S2).

Total YAP fluorescence intensity varied more than 10-fold across the screen (Figure 2B). YAP protein stability is regulated by the Hippo (Zhao et al., 2010) and Ras pathways (Hong et al., 2014), but we also found differences in YAP mRNA expression in some knockdowns (Figure S3), indicating regulation at the transcriptional or post-transcriptional level (Liu et al., 2010). Only wells with total intensities greater than −2 SD of control cells were counted as hits, because target gene expression did not correlate with high ratios in cells with low total YAP (Figure S3). Similarly, only wells with total intensities less than +2 SD of controls were counted as hits. By these criteria, 23% of targets had lower and 3% had higher than expected YAP ratios. The differences in total protein levels in YAP across the screen highlight the need to account for many sources of variation when interpreting imaging data.

### β-PIX and Associated Proteins Are Potential YAP Activators

This strategy identified both known and novel YAP regulators. Figure 2C shows average cell morphologies and subcellular YAP intensities represented as glyphs (PhenoPlot) (Sailem et al., 2015), plotted by the number of cells per well (x axis) and YAP_diff_ (y axis). LATS1 and LATS2 knockdown cells had high YAP_diff_, consistent with their known inhibitory functions (Figure 2C). Depletion of Cdc42, Rac1, the Rac1/Cdc42 GEF β-PIX (Cool1/ARHGEF7), and the Rac1/Cdc42 effector kinases PAK2 and PAK4 resulted in low YAP_diff_, suggesting that these targets are YAP activators. Cdc42 has previously been implicated as an upstream activator of YAP in the mammalian kidney (Reginensi et al., 2013, Huang et al., 2016) and the pulmonary alveolar epithelium (Liu et al., 2016). Rac1-TRIO signaling has been reported to drive YAP-TEAD association and target gene expression in uveal melanoma (Feng et al., 2014) and in *Drosophila* eyes (Jang et al., 2016). β-PIX was previously reported to be a negative regulator of YAP in breast cancer cells (Heidary Arash et al., 2014) and in fly (Dent et al., 2015) via GTPase-independent scaffold functions. However, our data indicate that β-PIX-mediated signaling contributes to YAP activation in normal breast myoepithelial cells. RhoA, RhoE, ROCK2, and focal adhesion kinase (FAK) (PTK2) knockdown cells also had low total YAP intensities, suggesting that cell-ECM adhesion proteins may also regulate YAP expression and/or stability.

**Figure 3:**
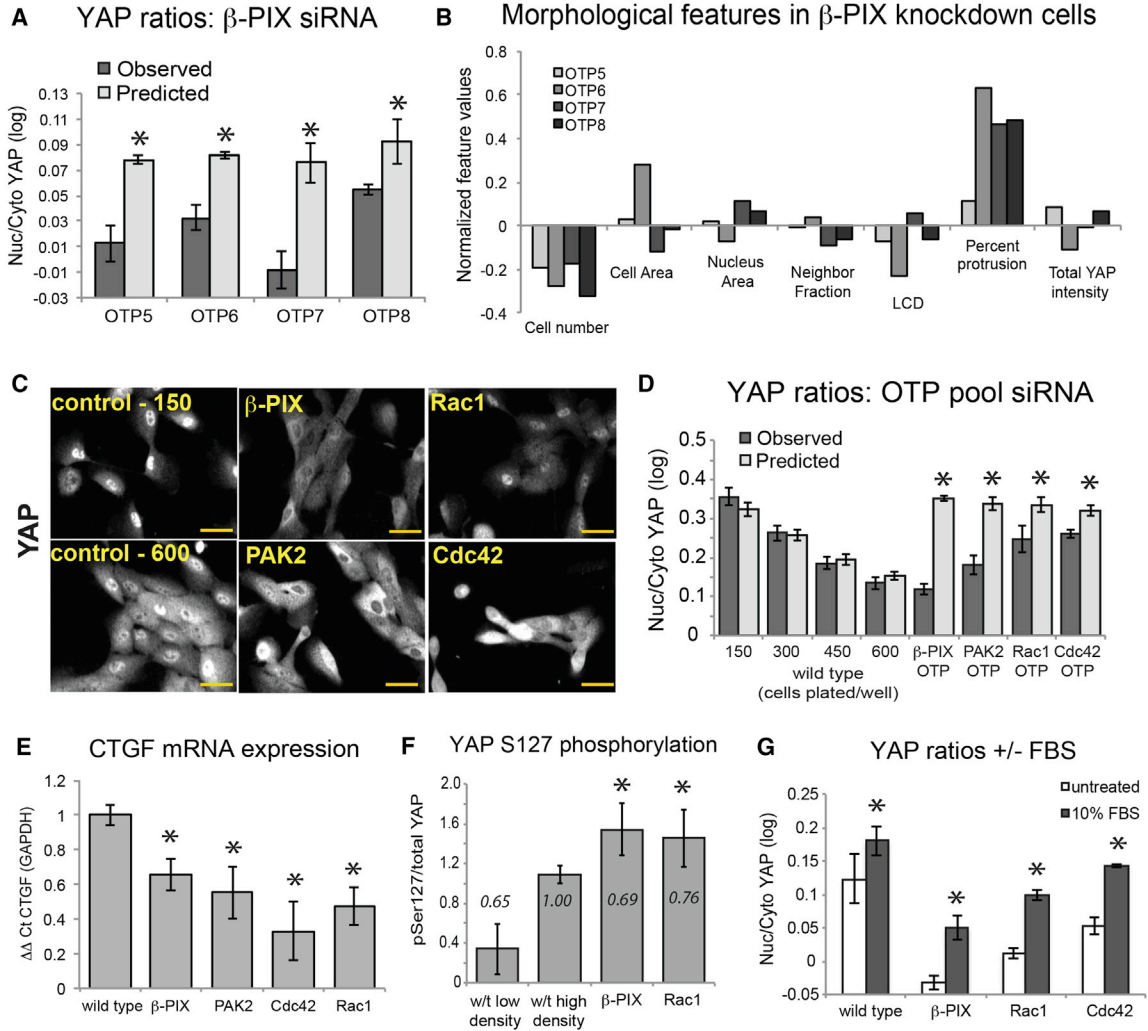
Validation of β-PIX, Rac1, Cdc42, and PAK2 as Regulators of YAP Nuclear Localization and Activation (A) Observed and predicted nuclear/cytoplasmic YAP ratios for cells transfected with siRNAs targeting β-PIX. Mean ± SD for replicate wells (n > 1,000 cells/well). ^∗^p < 0.001. (B) Average normalized morphological feature values. (C) Representative images of YAP staining. Scale bar, 20 μm. (D) Observed and predicted nuclear/cytoplasmic YAP ratios for wild-type wells seeded with the indicated numbers of cells per well or transfected with pooled siRNA. Mean ± SD (n > 1000 cells/well.) ^∗^p < 0.001. (E) Relative levels of *CTGF* mRNA normalized to *GAPDH* for wild-type and knockdown cells transfected for 48 hr, then seeded at the same low density on plastic for 24 hr. Mean ± SD (n = 3). ^∗^p < 0.01. Nuclear/cytoplasmic YAP ratios in cells stimulated with 10% FBS for 1 hr. ^∗^p < 0.01. (F) Ratio of phospho-S127 YAP to total YAP by western blot for low-density and high-density wild-type (w/t) and siRNA-transfected cells. Mean ± SD (n = 2). Relative number of cells/flask in italics. (G) YAP ratios in cells treated with DMEM alone or DMEM + 10% FBS (final concentration). ^∗^p < 0.01.

Gene depletion was verified by qRT-PCR (Figure S4), and on-target effects on YAP were validated using different siRNAs (Dharmacon OnTarget Plus; OTP) (Figures 3A and 3C; Figures S4 and S5B). Individual siRNAs targeting *ARHGEF7* all led to low YAP_diff_, despite having various effects on cell shape (Figure 3B). The effect of β-PIX depletion on YAP was also confirmed using another antibody, which binds the C terminus of YAP/TAZ (Figure S5A). Expression of the YAP/TAZ target gene *CTGF* was lower in all knockdown cells than in wild-type cells seeded at the same subconfluent density (Figure 3E).

To gain insight into how β-PIX regulates YAP, we measured phosphorylation of Ser127, a LATS1/2 (Hao et al., 2008, Oka et al., 2008) and Akt (Basu et al., 2003) target site. Subconfluent β-PIX and Rac1 knockdown cells had significantly more phospho-Ser127 YAP than even confluent wild-type cells (Figure 3F), suggesting that β-PIX and Rac1 affect the activity of Ser127 kinases and/or phosphatases (Qi et al., 2015). β-PIX, Rac1, and Cdc42 are not critical for YAP activation, however, as their depletion did not inhibit YAP nuclear translocation in response to fetal bovine serum (FBS) (Yu et al., 2012) (Figure 3G). Furthermore, YAP localization was sensitive to cell density in all knockdowns (Figure S5D), which suggests that Rac1, Cdc42, PAK2, and β-PIX are not required for inhibition of YAP by crowding.

### β-PIX Depletion Impairs Focal Adhesion Remodeling, but Not Protrusion Formation

**Figure 4:**
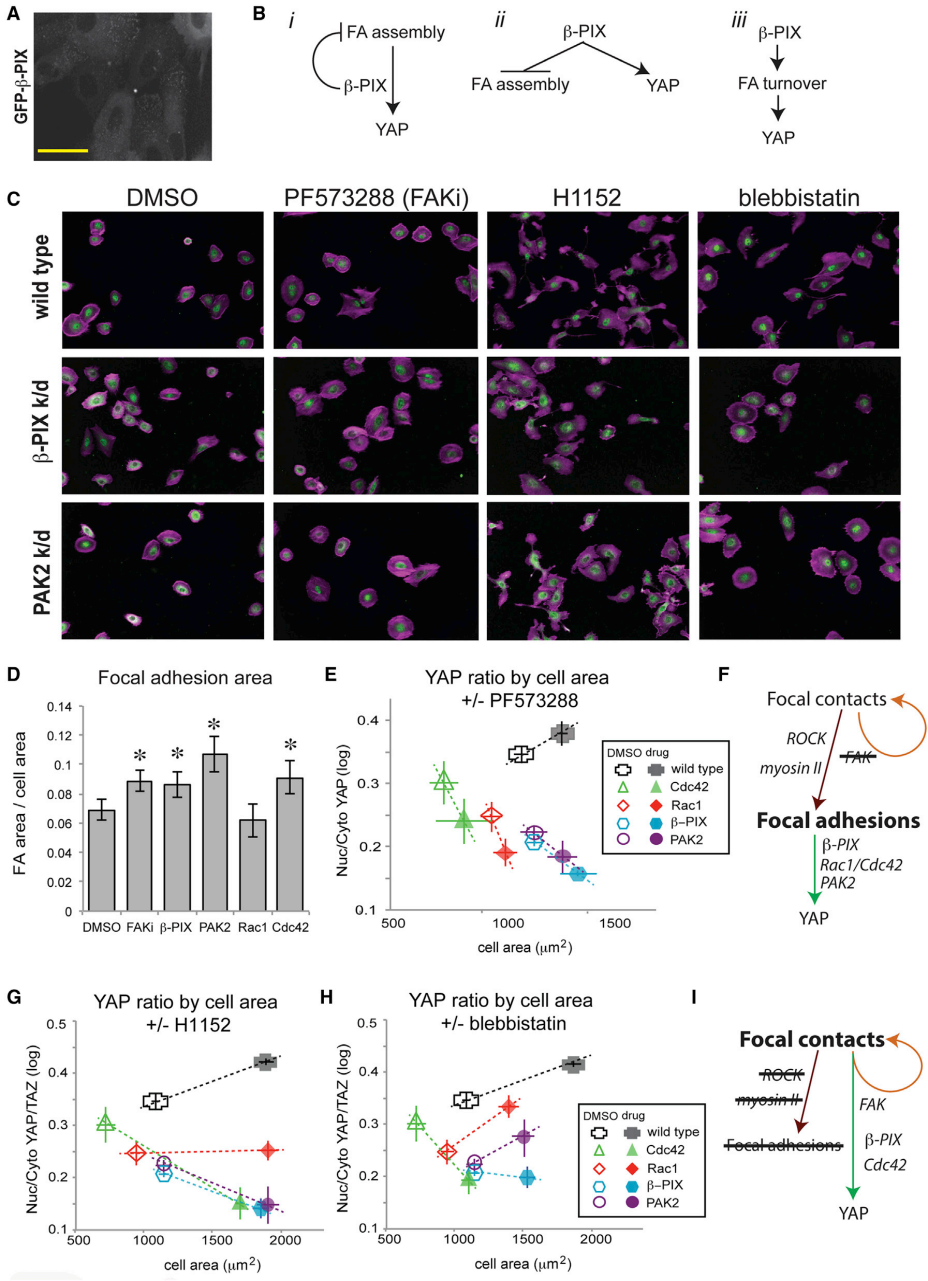
YAP Is Activated by β-PIX through Focal-Adhesion-Dependent and Focal-Adhesion-Independent Pathways during Cell Spreading (A) GFP-β-PIX in wild-type MCF10A cells. Scale bar, 20 μm. (B) β-PIX may couple YAP activation to focal adhesions (i), regulate YAP independently of focal adhesions (ii), or activate YAP indirectly by promoting focal adhesion turnover (iii). (C) Cells plated on fibronectin (FN) in the presence of DMSO (control) or small-molecule inhibitors of FAK (PF573288), ROCK (H1152), and myosin II (blebbistatin). Green, YAP; purple, F-actin. Scale bar, 20 μm. (D) Focal adhesion area (normalized to cell area) in cells plated on FN for 4 hr. Mean ± 95% CI (n = 30–80 cells/condition). (E) Nuclear/cytoplasmic YAP ratio as a function of cell area in cells plated on FN (4 hr) in the presence of DMSO or FAKi (PF-573288). Mean ± SD of replicate wells (n = 4 wells/condition, 230 ± 95 cells/well). (F) Inhibiting FAK kinase activity blocks turnover of focal contacts and promotes maturation and growth of focal adhesions. FAK-independent YAP activation requires β-PIX, Cdc42, Rac1, and PAK2. (G and H) Nuclear/cytoplasmic YAP ratio as a function of cell area in cells plated on FN (4 hr) in the presence of DMSO (open shapes) or inhibitors of ROCK (G) or myosin II (H) (filled shapes). Mean ± SD of replicate wells (n = 4 wells/condition, 230 ± 95 cells/well). (I) Inhibiting ROCK kinase or myosin II ATPase activity blocks focal adhesion maturation and promotes contact formation. Myosin-II-independent YAP activation requires β-PIX and Cdc42.

Although β-PIX knockdown resulted in decreased YAP nuclear translocation, its depletion resulted in morphological changes that would be expected to activate YAP. β-PIX knockdown cells were highly protrusive, which was positively correlated with YAP ratio in wild-type cells, although their migration speed and directional persistence were impaired (Figures S5F–S5H). They also had more and longer cell-ECM adhesions than wild-type cells (Figures S5I–S5K). β-PIX is recruited to nascent focal contacts and mature focal adhesions (Figure 4A) through association with Git1/2 and paxillin, where it triggers adhesion turnover (Feng et al., 2004, Kuo et al., 2011, Zhao et al., 2000). β-PIX could therefore be involved in transmitting signals from cell-ECM adhesions to YAP in addition to promoting focal adhesion turnover (Figure 4B, i). Alternatively, β-PIX could activate YAP independently of its role in focal adhesion remodeling (Figure 4B, ii), or, impaired YAP activation could be the result of to impaired focal adhesion dynamics (Figure 4B, iii).

### β-PIX Couples YAP Nuclear Localization to Cell Spreading

Cell spreading is a dynamic process that involves cycles of actin-driven protrusion and focal contact formation, followed by actomyosin-driven retraction and focal adhesion maturation or turnover (Parsons et al., 2010). To distinguish among the three possibilities shown in Figure 4B, we examined YAP localization in wild-type and knockdown cells spreading on FN with or without small-molecule inhibitors that alter focal adhesion dynamics. To minimize complicating effects of cell-cell contact, cells were plated at low densities where mean NF was less than 0.3. Cells were fixed 2–4 hr after plating, while the majority of cells were adherent but still actively spreading (Figure S7).

FAK plays a key role in focal adhesion turnover by transiently inactivating ROCK (Ilić et al., 1995, Webb et al., 2004). We therefore asked whether promoting focal adhesion maturation by inhibiting FAK had the same effect on YAP as β-PIX depletion. Short treatments with the FAK kinase inhibitor (FAKi) PF-573288 (2 μM) reduced FAK tyrosine phosphorylation (Figure S7A) and did not significantly affect YAP protein levels over this timescale (Figures S3E and S3F). FAK inhibition led to increased focal adhesion area (Figure 4D; Figure S8A) and actomyosin contractility (Figure S7B). Wild-type cells plated with FAKi were larger in area than cells plated with DMSO (control) (p < 0.01) (Figure 4E, x axis). The more rapid increase in cell area with FAKi (Figure S7A) was likely due to the fact that more nascent focal contacts could mature, anchoring each successive round of protrusion to the ECM and preventing retraction. YAP ratios were also higher in cells plated with FAKi than in controls (p < 0.01) (Figure 4E, y axis). YAP ratios tended to increase more sharply with cell area during spreading in the presence of FAK inhibitor (p < 0.01) (Figure S7E). Because the number of focal adhesions increases with cell area, this is consistent with the hypothesis that inhibiting FAK leads to focal adhesion maturation and/or mechanical force on focal adhesions, which then promotes YAP activation.

β-PIX, PAK2, and Cdc42 (but not Rac1) knockdown cells formed more focal adhesions than wild-type cells (p < 0.002) (Figure 4D). Cells lacking Cdc42 and Rac1 were smaller in area, consistent with the fact that these proteins are important for protrusion formation (Nobes and Hall, 1995), but β-PIX and PAK2 knockdown cells were comparable in area to controls. However, YAP ratios were significantly reduced in all knockdowns. Thus, depletion of β-PIX and PAK2 appears to uncouple YAP activation from cell spreading.

β-PIX and PAK2 knockdown cells plated with FAKi were significantly larger in area than knockdown cells plated with DMSO (p < 0.01) (Figure 4E, x axis), but Rac1 and Cdc42 knockdown cells were not. This supports the notion that the enhanced spreading seen in FAK-inhibited cells required Rac1/Cdc42-driven protrusion. Unlike wild-type cells, all knockdown cells plated with FAKi had lower YAP ratios than cells plated with DMSO (p < 0.001) (Figure 4E, y axis). Although FAK inhibition had a similar effect on focal adhesion dynamics to β-PIX, Cdc42, and PAK2 depletion, it had the opposite effect on YAP activation. These data support the hypothesis that β-PIX-Rac1/Cdc42-PAK2 signaling pathways activate YAP downstream of mechanically active focal adhesions, in which FAK kinase is not required (Figure 4F).

### YAP Is Activated through Both FAK-Independent and Myosin II-Independent Pathways

Adhesion complex maturation requires F-actin retrograde flow and myosin II (Stricker et al., 2013). If inhibiting FAK in spreading cells promoted YAP activation downstream of focal adhesions, we hypothesized that inhibiting actomyosin contractility would mitigate this effect. To test this, we plated cells with FAKi and/or ROCK inhibitor (H1152, 5 μM), which promotes Rac1-driven protrusion, focal contact formation, and focal adhesion turnover (Tsuji et al., 2002). ROCK-inhibited cells did not form large adhesions, but instead formed small peripheral adhesions (Figure S7C). Plating wild-type cells in the presence of H1152, or the ROCK inhibitor Y-27632 (Y27; 10 μM), led to increased cell areas and YAP ratios (Figure 4G; Figure S7). This is consistent with reports that YAP can be regulated independently of focal adhesions and actomyosin during cell spreading (Das et al., 2016, Zhao et al., 2012). Indeed, cells plated with H1152 tended to have more nuclear YAP than comparably sized control cells (p < 0.01) (Figure S7E). Plating cells with both FAKi and H1152 together mitigated the effect of either inhibitor alone (Figures S7D and S7E). This supports that idea that FAK-independent YAP activation requires ROCK kinase and also suggests that ROCK-independent YAP activation requires FAK kinase. We therefore propose that YAP can be activated during adhesion and spreading by two mechanisms: a FAK-independent, ROCK-dependent pathway that activated by focal adhesion maturation (Figure 4F) and a FAK-dependent, ROCK-independent pathway triggered by new ECM adhesions (Figure 4I), as reported by Kim and Gumbiner (2015).

### β-PIX and Cdc42 Are Required for Myosin-II-Independent YAP Activation

We next asked whether these proteins were involved in ROCK/myosin II-independent YAP activation. Plating cells with H1152 led to significant increases in cell area in wild-type and all knockdown cells (p < 0.001), but the YAP ratio only increased concomitantly with spreading in wild-type cells (p < 0.001) (Figure 4G). ROCK can antagonize Rac1 through multiple pathways (Guilluy et al., 2011), so we also tested the effect of blebbistatin (Blebb; 6 μM), which inhibits myosin II, to distinguish the roles of ROCK as a myosin activator versus a Rac1 inhibitor. All cells plated with Blebb had many small peripheral focal contacts (Figure S8B) and were larger in area that controls (p < 0.001) (Figure 4H). YAP ratios increased with cell area in wild-type, Rac1, and PAK2 knockdown cells plated with Blebb (p < 0.001), but not in Cdc42 or β-PIX knockdown cells (Figure 4H). The differences in the phenotypes of Rac1/PAK2 and β-PIX/Cdc42 knockdowns in the presence of ROCK and myosin II inhibitors suggest that YAP can be activated by distinct Rac1- and Cdc42-mediated pathways and that only β-PIX and Cdc42 are essential for myosin-independent YAP activation during cell spreading.

### The Effect of Kinase Inhibition on YAP Depends on Morphology and Microenvironmental Context

**Figure 5:**
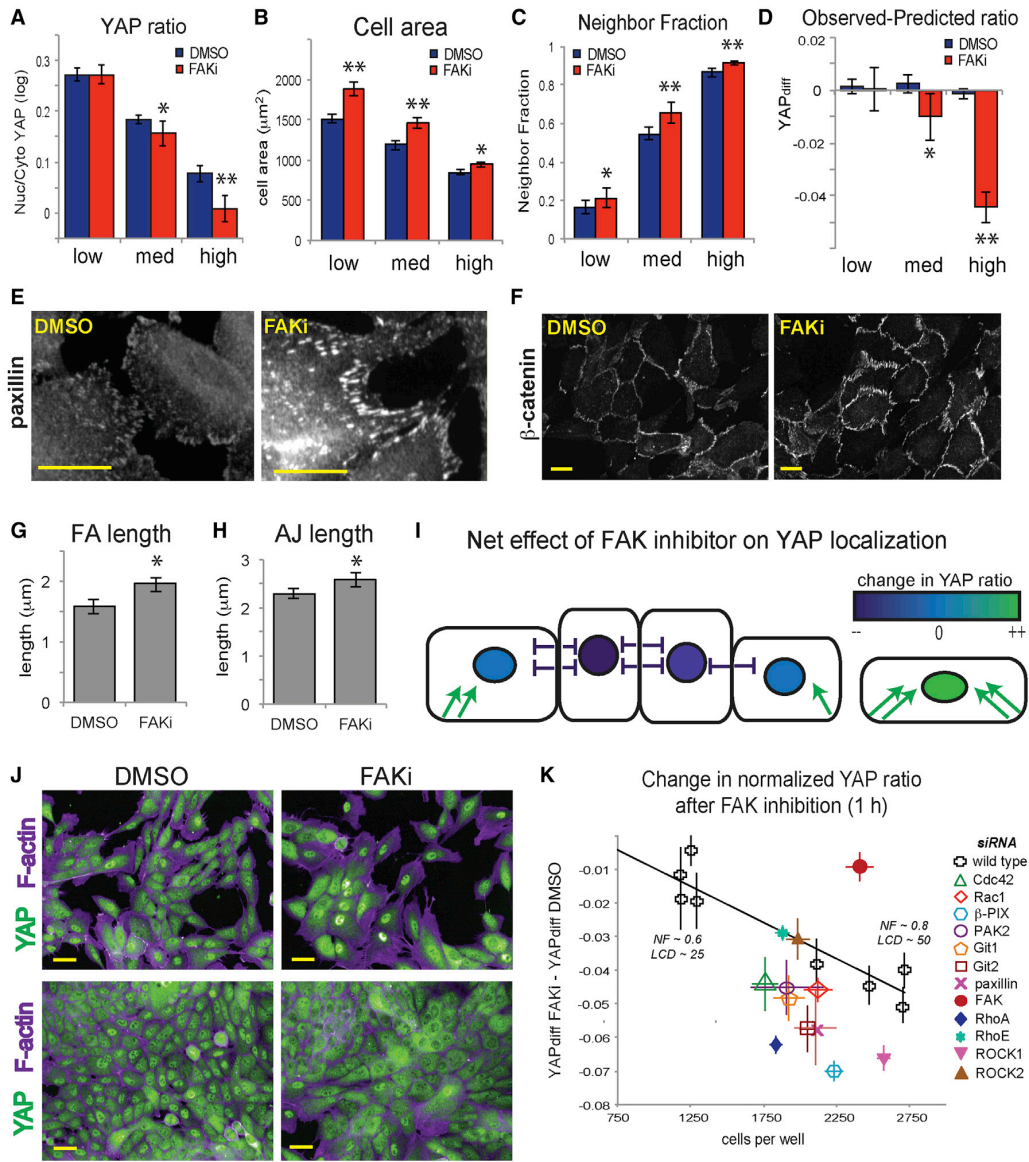
FAK Inhibition Can Have Positive and Negative Effects on YAP Nuclear Localization Depending on Cell-Cell and Cell-ECM Contact (A) Nuclear/cytoplasmic YAP ratios in low-, medium-, and high-density cells treated with DMSO or FAK inhibitor (PF-573288) for 1 hr. Mean ± SD (n = 8 wells/condition, 125–1,925 cells/well). ^∗^p < 0.01. ^∗∗^p < 0.001. (B) Difference between YAP ratios observed and predicted based on cell shape (YAP_diff_). ^∗^p < 0.05. ^∗∗^p < 0.001. (C) Cell area. Mean ± SD. ^∗^p < 0.05. ^∗∗^p < 0.001. (D) Neighbor fraction (NF). Mean ± SD. ^∗^p < 0.05. ^∗∗^p < 0.001. (E–G) Immunostained cells treated with DMSO or FAK inhibitor (FAKi). (E) Paxillin. Scale bar, 20 μm. (F) β-catenin. Scale bar, 20 μm. (G) Mean focal adhesion length ± 99% CI. (n = 500 adhesions/condition). (H) Mean adherens junction length (perpendicular to cell-cell border) ± 99% CI. (n = 1,000 adhesions/condition). (I) Activating signals predominate in low density or edge cells, whereas inhibitory signals predominate in crowded cells. At intermediate densities, positive and negative inputs can balance out, resulting in no “net” change in YAP localization. (J) Different fields of view taken from one DMSO-treated well (left) and one FAKi-treated well (right). Green, YAP; purple, F-actin. Scale bar, 20 μm. (K) Difference in shape-normalized YAP ratios between DMSO- and FAKi-treated cells. Mean ± SD (n = 4 wells/condition). Mean ± 95% CI for each wild-type well (n = 200–2,000 cells). Average NF and LCD for wild-type wells indicated.

Interpreting the effects of kinase inhibition on YAP in adherent cells presented a challenge, as drug treatments led to morphological changes associated with both YAP activation and inactivation. Cell area, which was positively correlated with the YAP ratio, increased upon FAK inhibition, particularly in low-density conditions where cells had more room to spread (Figure 5B; Figure S8B). Cell-cell contact, which was negatively correlated with the YAP ratio (Figure 5C), also increased in the presence of FAKi. Inhibiting FAK led to increased cell-cell adhesion length, as FAK kinase promotes adherens junction turnover (Playford et al., 2008) (Figures 5E and 5H). FAK may therefore activate or inactivate YAP indirectly via effects on cell-ECM and cell-cell adhesions (Figure 5I).

The effect of FAK inhibition on YAP depended on cell density (Figure 5A). Average YAP ratios increased in very sparsely plated cells treated with FAKi (NF < 0.2) (Figure S8A), were not significantly different in low- to medium-density wells, and decreased significantly in confluent (NF > 0.85) wells (Figure 5A). MLR analysis showed that YAP localization was consistent with shape changes at low densities, but not at high densities (Figure 5D). This may indicate that FAK kinase has other effects that are only apparent in crowded cells and/or that MLR models did not capture relevant morphological changes, such as growth of pre-existing adherens junctions. The heterogeneous effects of FAKi can be observed in different fields of view from the same well. FAKi-treated cells that were large and had few neighbors had more nuclear YAP than control cells, whereas those in crowded areas had less nuclear YAP (Figure 5J). Thus, the indirect activating and inhibitory effects of FAKi could effectively cancel out under certain conditions, such as intermediate densities, resulting in no “net effect” on YAP (Figure 5I).

FAK was reported to act upstream of Src-PI3K-PDK1 to inhibit LATS (Kim and Gumbiner, 2015). Inhibiting Src (PP2) or phosphatidylinositol 3-kinase (PI3K; LY294002) led to decreases in the YAP ratio at all densities, and neither Src nor PI3K inhibition had synergistic effects with FAK (Figures S8E–S8F). This suggests that Src and PI3K are critical components of YAP activation in response to adhesion but that FAK is dispensable where Src/PI3K can be activated through other pathways, such as epidermal growth factor receptor (EGFR) signaling.

Inhibiting actomyosin contractility had different effects on YAP in adherent and spreading cells. Inhibiting ROCK with Y27 (10 μM) appeared to have no effect on YAP localization (Figure S8C), but MLR analysis showed that YAP ratios were lower than expected, as Y27-treated cells were larger in area and had lower NF than controls (Figure S8D). Blebb treatment decreased YAP ratios in adherent cells at all densities (Figure S8C). FAKi led to decreases in YAP ratios in cells pretreated with Blebb or Y27, even at low densities (Figure S8C). This supports the hypothesis that FAK can indirectly inhibit YAP via its effects on ROCK/myosin II (e.g., by blocking focal adhesion maturation or actomyosin tension).

### β-PIX and Focal Adhesion Proteins Couple YAP to FAK-Inhibitor-Induced Morphological Changes

**Figure 6:**
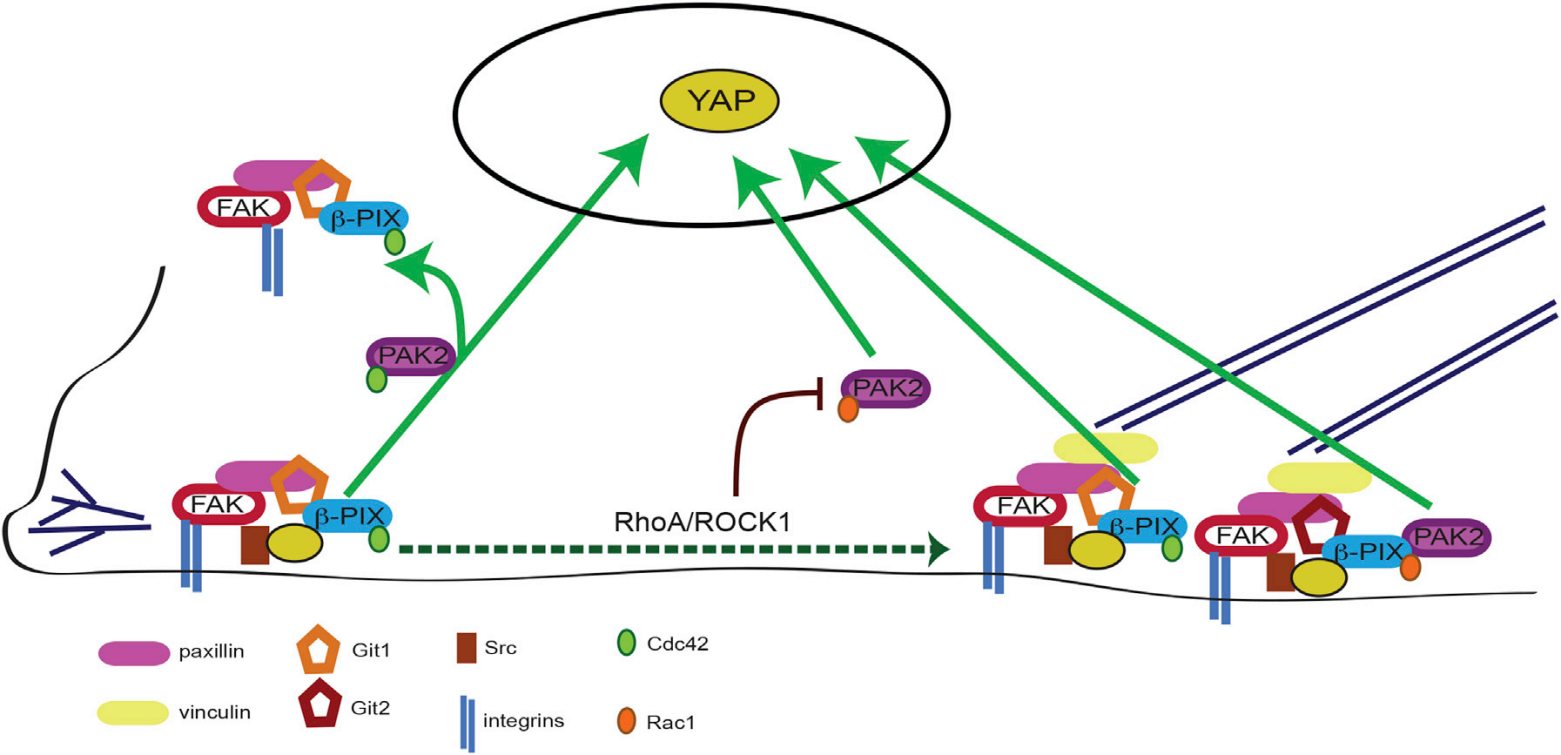
Model: β-PIX Couples YAP to Cell-ECM Adhesion Dynamics β-PIX is recruited to nascent focal contacts through association with Git1/2 and paxillin (left), where it is phosphorylated by Src. β-PIX can then activate Cdc42, triggering a negative feedback cycle of adhesion disassembly as well as FAK-dependent YAP activation. Newly formed focal contacts can mature into focal adhesions via RhoA/ROCK1/myosin II activity (right). YAP can then be further activated independently of FAK via β-PIX, Cdc42, Rac1, and PAK2. ROCK may also regulate YAP independently of myosin II by inhibiting Rac1/PAK2.

Next we asked whether the β-PIX-mediated signaling pathways we characterized in spreading cells play a role in YAP activation in response to changes in focal adhesion stability and actomyosin tension in adherent cells. We predicted that if a protein were involved in activating YAP in this pathway, then the combination of FAKi and gene depletion would have a synergistic effect on YAP (STAR Methods; Figure S9). That is, FAK inhibition would result in lower than expected YAP ratios in the knockdown cells because the activating signal would be lost. RhoA and ROCK1 depletion produced such synergistic effects with FAKi, as shown by the greater difference in normalized YAP ratios compared to control cells (Figure 5K), consistent with what we observed with ROCK inhibitor (Figure S8C). ROCK2 and RhoE, however, do not appear to be part of the actomyosin activation pathway, as YAP ratios in FAKi-treated cells changed predictably with cell shape in the absence of these proteins. RhoA and ROCK1 mediate focal adhesion maturation, whereas RhoE and ROCK2 have been implicated in driving adhesion turnover (Lock et al., 2012, Riento et al., 2005). β-PIX, Rac1, Cdc42, and PAK2 depletion had synergistic effects with FAK inhibition, as did depletion of Git1, Git2, and paxillin, which recruit β-PIX to adhesion complexes (Turner et al., 2001) (Figure 5K). From these data, we can infer that adhesion complexes are involved in activating YAP in response to actomyosin tension and/or focal adhesion maturation and that β-PIX signaling and small GTPases are essential components of this pathway (Figure 6).

### YAP Regulation by Cell-ECM Adhesion Is Not Conserved in Metastatic Breast Cancer Cells

**Figure 7:**
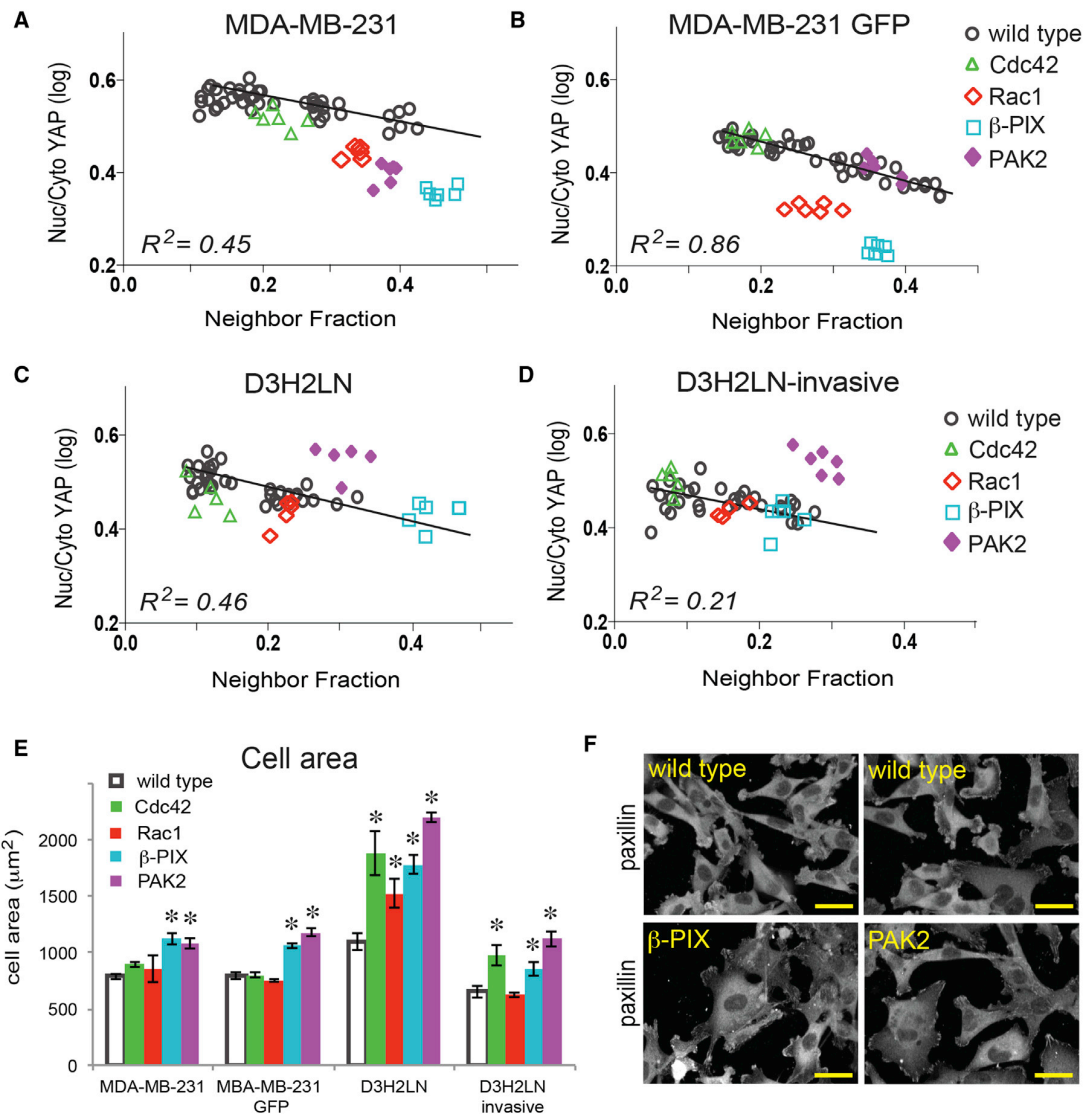
YAP Regulation Is Uncoupled from Cell-ECM Adhesion and β-PIX-Associated Genes in Metastatic Breast Tumor Cells (A–D) Average nuclear/cytoplasmic YAP ratios in replicate wells of wild-type (mock-transfected) and siRNA-transfected cells from four variants of MDA-MB-231 cells plotted as a function of cell-cell contact area (NF). (A) Parental cell population. (B) Parental cells selected to stably express GFP. (C) D3H2LN cells harvested from mouse lymph node metastasis. (D) D3H2LN cells selected by passage through 3 micron pores. Replicate well averages are shown (n = 150–2,500 cells/well). (E) Cell areas. Mean ± SD of replicate wells (n = 6 wells/condition). ^∗^p < 0.01 versus wild-type for each cell line. (F) Focal adhesions labeled by anti-paxillin immunofluorescence in representative fields of wild-type (top) and siRNA-transfected cells (bottom). Scale bar, 20 μm.

Finally, we examined cell shape and YAP localization in four variants of the triple-negative breast tumor cell line MDA-MB-231. One variant consisted of the parental line selected to stably express GFP. D3H2LN cells were derived from mouse lymph node metastasis of MDA-MB-231 (Jenkins et al., 2005), and a highly invasive variant of D3H2LN was generated by repeated harvesting of cells that traversed 3-μm pores. Although average YAP ratios were negatively correlated with average NF in all four lines (Figures 7A–7D, italics), correlation coefficients (*R*^*2*^) between the YAP ratio and NF were less than 0.1 at the single-cell level. Neither cell area nor A_nuc_/A_cell_ were correlated with YAP ratio in single cells (*R*^*2*^ < 0.01), and MLR analysis of MDA-MB-231 and D3H2LN cells using combinations of three to eight shape features (n > 25,000 cells) yielded *R*^2^ < 0.2. YAP nuclear localization is therefore largely uncoupled from cell-ECM contact and spreading, though not necessarily from cell density, in these triple-negative breast cancer cell lines.

Cdc42 depletion did not appear to affect YAP localization, though it did reduce cell numbers in all lines. Depletion of β-PIX and Rac1 led to decreased YAP ratios in the MDA-MB-231 (Figures 7A and 7B), but not in D3H3LN variants (Figures 7C and 7D). Although the average YAP ratios in β-PIX knockdown D3H2LN cells were slightly lower than controls, this difference could be accounted for by the increase in NF, which was correlated with YAP ratios on average (Figure 7C). Loss of PAK2 only reduced YAP ratios in one MDA-MB-231 line and actually increased YAP ratios in both D3H2LN cell lines (Figures 7C and 7D). All β-PIX- and PAK2-depleted cells were significantly larger in area and formed long focal adhesions, consistent with impaired adhesion remodeling (Figures 7E and 7F). Taken together, these data indicate that YAP activation is independent of Cdc42 in MDA-MB-231 cells and that β-PIX/Rac1/PAK2-mediated YAP regulation is abnormal in the most highly invasive cells. Uncoupling YAP activation from β-PIX signaling may allow tumor cells to sustain high levels of YAP activation in the absence of stable cell-ECM adhesions.

## Discussion

Using multiparametric analysis of single-cell morphology to score hits in RNAi screens based on relationships between measured variables, we identified Cdc42 and Rac1, the Cdc42/Rac1 GEF β-PIX, and the Cdc42/Rac1 effector kinase PAK2 as key mediators of YAP activation. Statistical models have previously been used to investigate the mechanisms that underlie cell behavior as a function of population context (Snijder et al., 2009, Snijder et al., 2012). Our approach differs from these studies in two ways. First, we used multivariate linear regression models rather than multidimensional binning to deal with continuous variables. Second, we used only morphologically heterogeneous wild-type cells, which have functional YAP regulatory machinery, to generate models. This allowed us to “normalize” for differences in cell morphology at the single-cell level and compare phenotypes across disparate populations.

Although linear regression is not ideal for complex systems with co-linear variables, or when the impact of one variable depends on the values of others, it is a simple but effective starting point to normalize data when perturbations may have both direct and indirect effects. More sophisticated machine learning techniques will help improve models and predictions in future studies. Shape-based models could also be improved by incorporating information about the cytoskeleton, adhesion distribution, and cell-cycle stage.

Multivariate analysis of genetic and chemical perturbations indicates that YAP can be activated through multiple mechanisms during cell spreading (Figure 6). As cells spread, new focal contacts are formed at the protrusive edges. Our data indicate that YAP is activated downstream of cell spreading through a pathway that is ROCK kinase/myosin II independent but requires FAK kinase, as well as a FAK-dependent signaling pathway downstream of nascent focal contacts. Further study is needed to distinguish the role of adhesion complexes in activating YAP during spreading, as plasma membrane distortion, endocytosis, and cytoskeletal reorganization may also be involved. The effect of ROCK and myosin II inhibition on YAP localization differed between spreading and adherent cells, as shown by Dupont et al. (2011), which is consistent with a recent report by Das et al. (2016) (Figure 4; Figure S8). Adherent cells already have nuclear YAP and experience a loss of cytoskeletal tension and ECM adhesion when treated with H1152 or blebbistatin. Cells plated after trypsinization start out with only cytoplasmic YAP, which can be activated by integrin ligation; for example, through FAK/Src-PI3K-PDK1 (Kim and Gumbiner, 2015), by F-actin remodeling (Das et al., 2016, Dupont et al., 2011), by tension on focal adhesions, or through other mechanisms. We found that β-PIX and Cdc42 were required for myosin-II-independent YAP activation in spreading cells, whereas Rac1 and PAK2 were also involved in nuclear localization of YAP in spreading cells plated with ROCK inhibitor. This suggests that Rac1 and PAK2 play roles in YAP activation that are distinct from actomyosin contractility and can be inhibited by ROCK.

As cells spread, some focal contacts disassemble while others are stabilized and mature into focal adhesions (Parsons et al., 2010). YAP activation in response actin contractility and/or focal adhesion maturation did not appear to require FAK kinase, because plating cells with FAK inhibitor led to increases in YAP ratio together with increases in cell area (Figure 4B), focal adhesion size (Figure 4E), stress fiber formation, and myosin II phosphorylation (Figure S7A). Plating cells with both FAK and ROCK inhibitors mitigated the effects of either drug alone, which suggests that inhibiting FAK can indirectly stimulate YAP via actomyosin (Figures S7D and S7E). Depletion of β-PIX, Cdc42, Rac1, and PAK2, as well as RhoA and ROCK1, reduced YAP activation in cells treated with FAK inhibitor, indicating that this signaling network couples YAP to mechanical signals. Furthermore, depletion of proteins that recruit β-PIX to adhesion complexes, Git1/2 and paxillin, also reduced YAP activation, supporting the hypothesis that this pathway is mediated by focal adhesion complexes.

In adherent cells treated with FAK inhibitor, YAP ratios increased only in cells with few cell-cell contacts (low NF) (Figure 5). In confluent cells, FAK inhibition led to significant decreases in YAP ratio that were not explained by the changes in cell shape included in MLR models. In addition to promoting adhesion turnover, FAK is involved in adherens junction turnover (Playford et al., 2008), and FAKi-treated cells showed increases in NF and cell-cell adhesion length (Figures 5F and 5H). FAK may thus activate YAP indirectly by promoting adherens junction turnover, as cell-cell adhesion promotes cytoplasmic retention of YAP through the Hippo pathway (Meng et al., 2016).

How might β-PIX-mediated small GTPase signaling contribute to YAP activation? Depletion of β-PIX and Rac1 led to increased Ser127 phosphorylation, which suggests involvement of LATS1/2, Akt, and/or serine phosphatases. Constitutively active Rac1 was also previously reported to induce dephosphorylation of YAP (Zhao et al., 2012). β-PIX was reported to bind Lats1 and YAP but, in contrast to our findings, was proposed to inhibit YAP activation through a GTPase-independent mechanism (Heidary Arash et al., 2014). However, the same protein can have different functions depending on its subcellular context. The GEF activity of β-PIX requires Src-dependent tyrosine phosphorylation, which can occur at cell-ECM adhesions, whereas the cytosolic protein has little GTPase activating ability (Feng et al., 2006). In addition to Lats inhibition by PDK1 (Kim and Gumbiner, 2015), Src and PI3K could activate YAP via β-PIX. Indeed, Src, Yes, and PI3K subunits were also hits in our screen (Data S2). Further study of the molecular mechanisms that link small GTPase signaling to YAP are needed to determine whether cell-ECM adhesions regulate YAP through Hippo-dependent or Hippo-independent pathways and to investigate the relationships between adhesion complexes and cell-surface receptors such as G-protein coupled receptors (GPCRs) (Yu et al., 2012).

β-PIX is involved in a negative feedback loop of cell-ECM adhesion turnover (Chang et al., 2007, Kuo et al., 2011, ten Klooster et al., 2006, Zhao et al., 2000). By coupling YAP nuclear translocation to cycles of adhesion remodeling, cells could rapidly and dynamically fine-tune gene expression in response to physical signals. This would establish a mechanism in which YAP activation is tightly linked to or timed with cell-ECM adhesion dynamics. Such mechanically driven timing may be important to scale YAP activation proportionally to the strength and/or number of adhesions.

Uncoupling YAP from adhesion signals could aid anchorage-independent growth, as seen in MDA-MB-231 breast cancer cells, which showed no correlation between cell area and YAP ratio. MDA-MB-231 cells are known to have abnormal Merlin/NF2 (Dupont et al., 2011, Shapiro et al., 2014), a Hippo pathway tumor suppressor that is a target of PAK2 (Kissil et al., 2002). Depletion of β-PIX, Rac1, and PAK2 had inconsistent effects on YAP in more metastatic MDA-MB-231 variants. This may point to a vulnerable nexus through which YAP is misregulated in cancer.

These studies demonstrate the importance of considering the complex relationships among cell and tissue geometry, external and internal mechanical forces, and overlapping or contradictory signal transduction when trying to dissect out signaling pathways and molecular mechanisms.

## STAR★Methods

### Key Resources Table

**Table:** 

REAGENT or RESOURCE	SOURCE	IDENTIFIER
**Antibodies**		

YAP/TAZ [67.3] (mouse)	Santa Cruz	Cat: sc-101199; RRID: AB_1131430
YAP/TAZ (rabbit)	Novus	Cat: NB600-220
Phospho-serine 127 YAP/TAZ (rabbit)	Cell Signaling	Cat: 4911S; RRID: AB_2218913
Paxillin (mouse)	BD Transduction Labs	Sero et al., 2011
Paxillin [Y113] (rabbit mAb)	Abcam	Cat: ab32084; RRID: AB_779033
FAK (rabbit)	Cell Signaling	N/A
Phospho-Y397 FAK (rabbit)	Abcam	Cat: ab39967; RRID: AB_955850
β-catenin (mouse)	Cell Signaling	Cat: 610153
Phospho-myosin II light chain [519] (rabbit)	Cell Signaling	Cat: 3671P; RRID: AB_10859887
GAPDH [1D4] (mouse)	Novus	Cat: NB300-221
Vinculin (mouse)	BD Transduction Labs	Sero et al., 2011
Alexa 488/647 goat anti-mouse IgG (H+L)	Invitrogen (Thermo)	Cat: A11029, A21235; RRID: AB_141693
Alexa 488/647 goat anti-rabbit IgG (H+L)	Invitrogen (Thermo)	Cat: A21121, A21244; RRID: AB_141663
DyLight anti-mouse IgG (H+L) 680	Cell Signaling	Cat: 5470S; RRID: AB_10696895
DyLight anti-rabbit IgG (H+L) 800	Cell Signaling	Cat: 5151S; RRID: AB_10697505

**Chemicals, Peptides, and Recombinant Proteins**		

DMEM:F12 with GlutaMAX	GIBCO	Cat: 31331
Fetal Bovine Serum (heat-inactivated)	Sigma	Cat: F-9665
Insulin	Sigma	Cat: I-1882
Cholera toxin	Sigma	Cat: C-8052
Hydrocortisone	Sigma	Cat: H-0888
Human EGF	Sigma	Cat: E-9644
PF-573288	Tocris Biosciences	Cat: 3239; CAS 869288-64-2
H1152	Tocris Biosciences	Cat: 2414; CAS 871543-07-6
Blebbistatin	Sigma	Cat: B0560; CAS 856925-71-8
Y-27632	Sigma	Cat: Y0503; CAS 129830-38-2
LY294002	Sigma	Cat: L9908; CAS 154447-36-6
PP2	Calbiochem	CAS 172889-27-9
nocodazole	Sigma	Cat: M1404; CAS 31430-18-9
Fibronectin (bovine plasma 1 mg/ml)	Sigma	Cat: F1141
16% formaldehyde solution	Thermo	Cat: 28908
Alexa-568 phalloidin	Invitrogen (Thermo)	Cat: A12380
Lipofectamine RNAiMAX	Thermo	Cat: 13778038
Lipofectamine 2000	Thermo	Cat: 11668027
Trizol	Ambion	Cat: 15596026
RNeasy kit	QIAGEN	Cat: 74134

**Deposited Data**		

Image and analysis datasets	This paper	Image Data Repository accession number S-BSMS6 and the Biostudies database: https://www.ebi.ac.uk/biostudies/studies/S-BSMS6

**Recombinant DNA**		

pEGFP-C3-hYAP1	Basu et al., 2003	Addgene 17843
GFP-β-PIX	C. Waterman (NIH); Kuo et al., 2011	N/A
pEGFP-N1	Clontech	N/A

**Sequence-Based Reagents**		

siGENOME Smart Pool custom libraries	Dharmacon	See gene list or contact J.S.
ON-TARGETplus ARHGEF7	Dharmacon	L-009616
ON-TARGETplus RAC1	Dharmacon	L-003560
ON-TARGETplus CDC42	Dharmacon	L-005057
ON-TARGETplus PAK2	Dharmacon	L-003597
ON-TARGETplus RHOA	Dharmacon	L-003860
si-GENOME RHOA	Dharmacon	M-003860
ON-TARGETplus ROCK1	Dharmacon	L-003563
ON-TARGETplus ROCK2	Dharmacon	L-004610
ON-TARGETplus RND3 (RhoE)	Dharmacon	L-007794
ON-TARGETplus YAP1	Dharmacon	L-012200
ON-TARGETplus WWTR1 (TAZ)	Dharmacon	L-186083
siGENOME WWTR1 (TAZ)	Dharmacon	M-106083
siGENOME ARHGAP10	Dharmacon	M-009382
Predesigned siRNA: PXN	Ambion; Sero et al., 2011	Cat: 16708A; ID:118096
Predesigned siRNA: GIT1	Ambion	Cat:AM16104
Predesigned siRNA: PKL (Git2)	Ambion	Cat: AM51331, AM16708
Custom siRNA: FAK; GCGAUUAUAUGUUAGAGAUAGUU; CUAUCUCUAACAUAUAAUCGCUU	Dharmacon; Mammoto et al., 2007	N/A

**Experimental Models: Cell Lines**		

MCF10A human mammary gland cells	ATCC	CRL-10317
MDA-MB-231 human breast tumor cells	J. Erler (Copenhagen)	N/A
MDA-MB-231 GFP cells	C. Isacke (ICR)	N/A
D3H2LN cells (MDA-MB-231 mouse lymph node metastasis)	M. Olson (Beatson); Jenkins et al., 2005	N/A
D3H2LN-invasive cells	M. Olson (Beatson)	N/A

**Software and Algorithms**		

Acapella 4.0	PerkinElmer	N/A
Columbus Image Data Storage and Analysis System	PerkinElmer	http://www.cambridgesoft.com/ensemble/spotfire/Columbus/default.aspx
MATLAB	MathWorks	https://www.mathworks.com/
Vassar Stats	http://www.vassarstats.net	N/A
Prism	GraphPad	https://www.graphpad.com/scientific-software/prism/
Excel	Microsoft	N/A
Cluster 3.0	de Hoon et al., 2004	http://bonsai.hgc.jp/∼mdehoon/software/cluster/software.htm
ImageJ	Schneider et al., 2012	https://imagej.nih.gov/ij/

### Contacts for Reagent and Resource Sharing

Further information and requests for resources and reagents should be directed to and will be fulfilled by the Lead Contact, Julia Sero (juliasero@post.harvard.edu).

### Experimental Model and Subject Details

#### Cell Lines and Cell Culture

MCF10A cells obtained from ATCC were cultured in DMEM:F12 (GIBCO) containing 5% heat-inactivated fetal bovine serum (HI-FBS; GIBCO), 20 ng/ml EGF (Sigma), 10 μg/ml insulin (Sigma), 0.5 μg/ml hydrocortisone (Sigma) and 100 ng/ml cholera toxin (Sigma) at 37°C and 5% CO_2_, and cells used between passages 3 and 8.

MDA-MB-231 (Sero et al., 2015), and MDA-MB-231-GFP (Henry et al., 2011), cells were maintained in standard culture conditions (DMEM+10% FBS). D3H2LN cells are MDA-MB-231 variants harvested from mouse mammary fat pad metastases to the lymph node (Jenkins et al., 2005). D3H2LN-invasive cells were obtained by repeatedly harvesting D3H2LN cells that passed through 3 μm pores (courtesy Mike Olson, Beatson Institute, Glasgow).

All cell lines were confirmed to be mycoplasma-negative (LookOut mycoplasma PCR detection kit, Sigma-Aldrich). Passage was carried out using 0.25% trypsin-EDTA (GIBCO) followed by centrifugation (1000 rpm, 4 min) and resuspension in complete medium. Cell counting was performed using Countess automated cell counter with trypan blue exclusion (Thermo).

### Method Details

#### General Study Design for Image Analysis

Screens were performed using duplicate plates for both kinases (K) and GEFs/GAPs/GTPases (GGG). GGG plates contained two technical replicates per plate for each gene. At least 12 mock-transfected wells were included in each plate, and these wells were distributed so that they were not all in the same row or column. Pilot screens were performed three times using the GGG library, and both GGG and K libraries were re-screened and analyzed independently several months apart. All validation, drug treatment, and cell spreading experiments were conducted using at least 4 technical replicates (wells) per condition per experiment, and typically no less than 100 cells per well measured per well. At least two independent biological replicates were performed for all image analysis experiments, and only those in which the same trends were observed consistently over multiple experiments were included in the manuscript. Standard deviations of well averages are shown for technical replicates, and 99% confidence intervals are shown for means of single cell data.

#### RNAi Transfection, Fixation, and Staining

RNAi screens were performed in 384-well Cell Carrier plates (Greiner) to which 40 nl/well siRNA (20 μM) were plated using an Echo liquid handler (LabCyte). Prior to seeding cells, 10 μL of OptiMEM (GIBCO) containing 40 nl/well Lipofectamine RNAiMAX (Invitrogen) was added using a Multidrop Combi Reagent Dispenser (Thermo) and plates were incubated for 30 min at room temperature (RT). During this incubation, cells were harvested by trypsinization, counted and resuspended in 4/3X transfection medium. For all wells containing siRNA, and a subset of mock-transfected control wells, 600 cells/well were seeded in 30 μL of transfection medium (to give 1X final concentration of serum and growth factors). Mock-transfected wells in each plate were seeded with 150-450 cells/well for density controls. Importantly, the outer wells were filled with 50 μL sterile H_2_O or phosphate buffered saline (PBS) to prevent edge effects due to evaporation. Cells were incubated for 72 hr (unless otherwise indicated), and drugs (2 μM PF-573288, 5 μM H1152, 0.1 μg/ml nocodazole) or DMSO (final concentration 1:1000) were added 4 hr before fixation. Previously validated siRNA targeting *ECT2* and *PLK1* were used as transfection controls. siRNA transfection was verified by the presence of multinucleated cells for *ECT2* or cell death for *PLK1*. siRNA targeting *YAP1* was also included to confirm gene knockdown and antibody specificity.

Cells were fixed by adding 10 μL of pre-warmed 16% formaldehyde (Thermo) was added to each well by Multidrop and incubated for 15 min at RT. After washing 3X with PBS, cells were permeabilized in 0.1% Triton X-100. Primary antibodies were added in 10 μL PBS, outer rows and columns were topped up with 50 μL water or PBS, and plates were sealed and incubated overnight at 4°C. Following 3X washes in PBS, secondary antibodies (Alexa 488 and Alexa-647 conjugated anti-mouse and anti-rabbit; Invitrogen) and Alexa-568 phalloidin (Invitrogen) were added as above and incubated for 1 hr at RT. Plates were washed 2X in PBS, incubated for 10 min with 5 μg/ml Hoechst (Invitrogen), washed 1X, filled with 50 μL PBS, and sealed for imaging.

#### RNAi Validation, Cell Spreading, and Live Cell Imaging Experiments

All imaging experiments were performed using cells plated in 384-well Cell Carrier plates on Opera Cell::Explorer microscopes using culture, transfection, fixation, and staining protocols as described above. Gene knockdown validation experiments were performed using Dharmacon OnTargetPlus siRNA pools with the exception of previously validated custom siRNA targeting *FAK*/*PTK2* (Mammoto et al., 2007) and siRNA targeting paxillin, Git1, and Git2 (Ambion).

For cell spreading experiments, 384-well plates were coated with 1 μg/ml fibronectin (Sigma) and washed 2X with PBS followed by 1X with DMEM:F12 prior to seeding cells in complete medium containing drugs or DMSO. Live cells imaging of spreading cells was performed using cells labeled for 30 min with 10 μg/ml CellTracker Orange (Invitrogen) prior to trypsinization and re-plating on fibronectin-coated 384-well Cell Carrier plates.

Live cell imaging was performed on the Opera Cell::Explorer using a climate control chamber (37°C, 5% CO_2_, 75% humidity). pEGFP-C3-hYAP1 (GFP-YAP1) was a gift from Marius Sudol (Addgene plasmid 17843) (Basu et al., 2003), pEGFP-β-PIX was given by Clare Waterman (NHLBI, NIH, Bethesda, MD, USA), and pEGFP-N1 was from Clontech. Plasmids were transiently transfected with Lipofectamine 2000 (Invitrogen) for 4-6 hr, followed by overnight recovery, and GFP-positive cells were harvested by fluorescence activated cell sorting (FACS; BD FACSAria).

#### Quantification of GFP-YAP1 in Live Cells

MCF10A cells transiently transfected with GFP-YAP1 or GFP alone were imaged at 10 min intervals. The mean fluorescent intensities of whole cells were measured at each time point after addition of DMSO or FAK inhibitor. First, intensities at each time point were normalized to t = 0 for each cell. Then GFP-YAP1 measurements were normalized to the means of GFP measurements at each time point for each treatment group. This was necessary in order to control for differences in intensity resulting from changes in cell shape because FAKi-treated cells tended to become flatter and therefore dimmer over time.

#### High-Content Imaging

Image acquisition was performed using Opera Cell::Explorer automated spinning disk confocal microscopes. Screens were performed using a 20X air objective lens (NA = 0.45) (PerkinElmer) and 30 fields of view (checkerboard pattern) were imaged in each well. Other experiments were imaged using 20X water (NA = 0.7), 40X air (NA = 0.6), or 40X water (NA = 0.9) objective lenses. For all images from the same experiment shown together, exposure, contrast, and brightness settings were set identical parameters.

#### Cell Segmentation and Shape Feature Extraction

Automated segmentation was performed using Acapella (PerkinElmer). Nuclei were segmented using the Hoechst channel and the “nucleus region” was eroded by one pixel in order to compensate for segmentation errors (Figure S10A). Cell bodies were segmented using the YAP and actin channels as follows. First, cell cores were segmented using a watershed algorithm (Figure S10B). Then the cell core segments were used as seeds for a second watershed segmentation step performed on the actin channel (Figure S10C). The perinuclear or “ring region,” defined as a region encompassing two to seven pixels from the nucleus border, was used to measure cytoplasmic YAP intensity (Figure S10D). (one pixel = 0.64 μm at 20× magnification.) Nuclear/cytoplasmic YAP ratios were calculated as the log_10_ of the mean nuclear intensity/mean ring region intensity per cell. Nuclear/ring region intensity ratios were more reliable and robust than nuclear/whole cell or nuclear/cytoplasm ratios for cells with different shapes, as the ring region is most similar in thickness and focal plane to the nucleus region (Sero et al., 2015). Using log_10_ ratio is more appropriate for calculating mean and standard deviations than straight ratios, because values above 1 are equivalent in scale to values below 1.

Context features that describe the relationship of a cell to other cells were also measured. Neighbor fraction (NF) was determined as the proportion of a cell’s border in contact with other cells (Figure S10E). Local cell density (LCD) was measured by calculating the free space between nuclei by Voronoi segmentation. Cells within 30 pixels of the image border were excluded from analysis in order to use the LCD feature (Figure S10F).

Protrusions were determined by detecting regions in cytoplasm with intensity lower than 0.75 of average cytoplasm intensity based on the YAP channel to define the core of the cell body (Figure S10G). Protrusion extent was defined as the proportion of the border of the cell core in contact with protrusive regions.

Focal adhesion number and length were measured by hand using ImageJ (NIH). Focal adhesion area was determined from automatically segmented images using the Columbus (PerkinElmer) spot detection algorithm (D) and normalized to cell area (Figures S10I and S10J).

#### Filtering of Mitotic and Poorly Segmented Cells

Mitotic cells were filtered from the dataset using a combination of Hoechst intensity, nucleus area, nuclear roundness, nuclear width, and Hoechst channel pixel intensity distribution (to identify interphase nuclei containing nucleoli) (Figure S10H). Dead and poorly segmented cells were filtered based on ring region intensity, nucleus intensity, percent protrusion, cell area, nucleus area, and A_nuc_/A_cyto_. Segmentation parameters were defined and quality control was performed for a representative range of cells in each plate or experiment, particularly cells with extreme phenotypes (e.g., large area, irregularly shaped nuclei) and/or low YAP intensity.

#### Multivariate Linear Regression Analysis

To normalize YAP ratios for cell shape, multivariate linear regression (MLR) analysis was performed on wild-type, mock-transfected cells using MATLAB (Mathworks) or an online tool (http://www.vassarstats.net). The six features used in models were cell area (cellA), nucleus area (nucA), NF, LCD, total protrusion area (proA), and protrusion extent (proX), resulting in the following equation for each plate:

$$\text{YAP}\;\text{ratio}={\text{x}}_{1}\left(\text{cellA}\right)+{\text{x}}_{2}\left(\text{nucA}\right)+{\text{x}}_{3}\left(\text{NF}\right)+{\text{x}}_{4}\left(\text{LCD}\right)+{\text{x}}_{5}\left(\text{proA}\right)+{\text{x}}_{6}\left(\text{proX}\right)+\text{intercept}$$

Feature coefficients were similar across all ten plates in the screen, suggesting good reproducibility (Data S3), and models generated from control cells in two separate pilot screens showed similar coefficients (Data S3). Six-feature MLR models explained around 47% of the variation in YAP ratio (*R*^*2*^ = 0.4671 ± 0.015, SE 0.1309 ± 0.00687, p < 0.0001) and residuals were normally distributed around zero. The average prediction difference (YAP_diff_) for all control wells in the screen was −0.014 ± 0.236 and the average prediction error was 0.106 ± 0.007. Average y-intercepts and coefficients from 10 iterations of MLR performed on 500 randomly selected cells per plate were comparable to those derived from all control cells per plate, so for the sake of efficiency the entire set of control cells were used to generate MLR models. These models tended to slightly overestimate YAP ratios in high-density control wells (Figures 2C and S11C), but because most of the siRNA-treated wells were not in this range, we favored models that were more accurate at lower densities.

**Table:** 

	MLR6 Coefficient	MLR6 β	Average Coefficient (10 iterations)	Coefficient Std Dev (10 iterations)
Intercept	0.6145		0.6139	0.0405
Cell Area	3.242x10^−5^	0.2252	3.814x10^−5^	1.564x10^−5^
Local Cell Density	−3.201x10^−2^	−0.4345	−2.972x10^−2^	2.67x10^−3^
Neighbor Fraction	−0.1413	−0.2282	−0.1554	0.0566
Nuclear Area	−4.743x10^−3^	0.3449	−4.929x10^−3^	1.213x10^−3^
Protrusion Extent	−0.1357	−0.1988	−0.1423	0.0492
Protrusion Area	3.86x10^−5^	0.0342	2.43x10^−5^	8.95 x10^−5^
R-squared	0.4776			
root mean squared error	0.1054			

The coefficients of the multiple linear regression equations do not reflect their relative contributions because feature values were not scaled. The standardized regression weights (β) give an indication of the ranked importance of the features to the model. Features were not scaled because the ranges of some feature values were not normally distributed (Figure S11). The protrusion features proA and proX contributed the least to models; however, including these variables did increase the R^2^ (MLR6 = 0.4776; MLR4 = 0.4648) and decrease the root mean squared error of predictions (MLR6 = 0.1054; MLR4 = 0.1322), and were always highly significant (p < 0.001).

Linear regression models are not ideal for describing relationships between YAP and shape features, because of 1) co-linearity between features (Tables S1 and S2) differences in the contribution of features depending on magnitude or other variables. For example, YAP ratio was more highly correlated with nuclear area/cell area ratio (A_nuc_/A_cell_) in subconfluent cells (*R* = −0.397, n = 8753 cells; LCD < 50) than in confluent cells (*R* = −0.132, n = 17797 cells; LCD > 50).

#### Determination of Synergistic Effects between Gene Knockdown and FAK Inhibition

To dissect out the influences of morphology and gene depletion, YAP ratios were normalized by cell shape using MLR analysis, then the differences in normalized YAP ratios (YAP_diff_) between control (DMSO) and FAKi-treated wells were calculated (Figures 5K and S10). The change in YAP_diff_ was plotted as a function of cell number, and the distance from the best-fit regression line of wild-type cells (*R*^2^ = 0.88) indicates the magnitude of the FAK inhibitor effect in knockdowns (Figure 5K). In these experiments, cells were treated and fixed 48 hr after transfection, rather than 72 hr, to minimize differences in cell number and total YAP levels (Figure S9).

#### mRNA Quantification by qRT-PCR

Total RNA was extracted using phenol:cholorofom and isopropanol extraction (TRIzol) and the RNEasy Minikit (QIAGEN) according to the manufacturer’s protocol. 500 ng of purified RNA was converted to cDNA in a reaction containing 1mM dNTPs, 0.02 mM DTT, 1U MMLV, 2U RNase OUT (Invitrogen), and 0.3 mM random primers (Promega) for 90 min at 37°C. Quantitative real time polymerase chain reaction (qRT-PCR) was performed using cDNA made from extracted RNA using SYBRGreen PCR Master Mix (Invitrogen) and fluorescence was measured using on a 7300 AB system (Applied Biosystems). ΔΔCt quantitation was performed on triplicate absolute measurements and normalized to GAPDH or beta-actin mRNA (Figure S3). PCR primers are listed in Table S2. Quantitative RT-PCR for *CTGF*, *YAP1*, and *WWRT1/TAZ* expression were performed two to three times for each condition; representative experiments are shown. Gene knockdowns were determined one to two times per gene.

#### Western Blotting

Cells were harvested by trypsinization or by scraping in 1% NP-40/0.1% SDS lysis buffer (50 mM Tris 7.4, 150 mM NaCl, 1mM EDTA) with protease and phosphatase inhibitor cocktail (Thermo). Western blots were performed using 10% Tris-Glycine SDS-PAGE gels or 4%–20% Tris-HEPES gels. Proteins were transferred to PVDF membranes and incubated overnight at 4°C with primary antibodies. Dylight 800 anti-rabbit and 680 anti-mouse fluorescent secondary antibodies (Cell Signaling) were used to visualize bands on an Odyssey imager (Li-Cor). Phospho-S127 YAP and total YAP were visualized simultaneously on the same blots. Quantification of band intensities was performed using ImageJ. Means of technical replicates from representative experiments are shown.

#### Cell Cycle Analysis

Cells cultured in 6-well plates were transfected with siRNA for 72 hr prior to harvesting by trypsinization. Cells in suspension were washed 1X with PBS and fixed with 10% cold ethanol. DNA content was determined by labeling with Hoechst (Invitrogen) and quantified by FACS (BD FACSCalibur).

### Quantification and Statistical Analysis

*P*-values (Student’s t test) and confidence intervals were determined using Excel. *R* and *R*^*2*^ values (Pearson’s correlation) were determined using Excel, MATLAB (MathWorks), and Vassar Stats (http://www.vassarstats.net). Best-fit lines used for normalization of features were determined using Excel. Analysis of regression lines was performed with Prism (GraphPad). Principal component analysis (PCA) was performed for 15 shape features using Cluster 3.0.

### Data and Software Availability

Image and analysis datasets are available from the Image Data Repository (http://idr-demo.openmicroscopy.org/about, accession number S-BSMS6) and the Biostudies database (https://www.ebi.ac.uk/biostudies/studies/S-BSMS6).

## Author Contributions

J.E.S. designed and conducted the experiments, analyzed the data, and wrote the manuscript. C.B. provided support, discussion, and editing throughout.

## Supplemental Information

Document S1. Figures S1–S11 and Tables S1 and S2

Data S1. Related to Figure 2 Gene targets that scored as hits for YAP localization inconsistent with cell shape in two independent screens.

Data S2. Related to Figure 2 Normalized feature values for each gene target (average and SD for replicate wells).

Data S3. Related to Figure 2 and STAR Methods Multiple linear regression analysis coefficients for GGGK screen and repeat plates.

Document S2. Article plus Supplemental Information
