# Supplementary material for: Multiparametric Analysis of Cell Shape Demonstrates that β-PIX Directly Couples YAP Activation to Extracellular Matrix Adhesion
Source: Cell Syst. 2017 Jan 25;4(1):84–96.e6. doi: 10.1016/j.cels.2016.11.015 (PMC5289939; doi:10.1016/j.cels.2016.11.015)
Supplement: Document S1. Figures S1–S11 and Tables S1 and S2 [file mmc1.pdf]

Cell Systems, Volume *4*

Supplemental Information

Multiparametric Analysis of Cell Shape Demonstrates that β-PIX Directly Couples YAP Activation to Extracellular Matrix Adhesion

Julia E. Sero and Chris Bakal

Supplementary Information

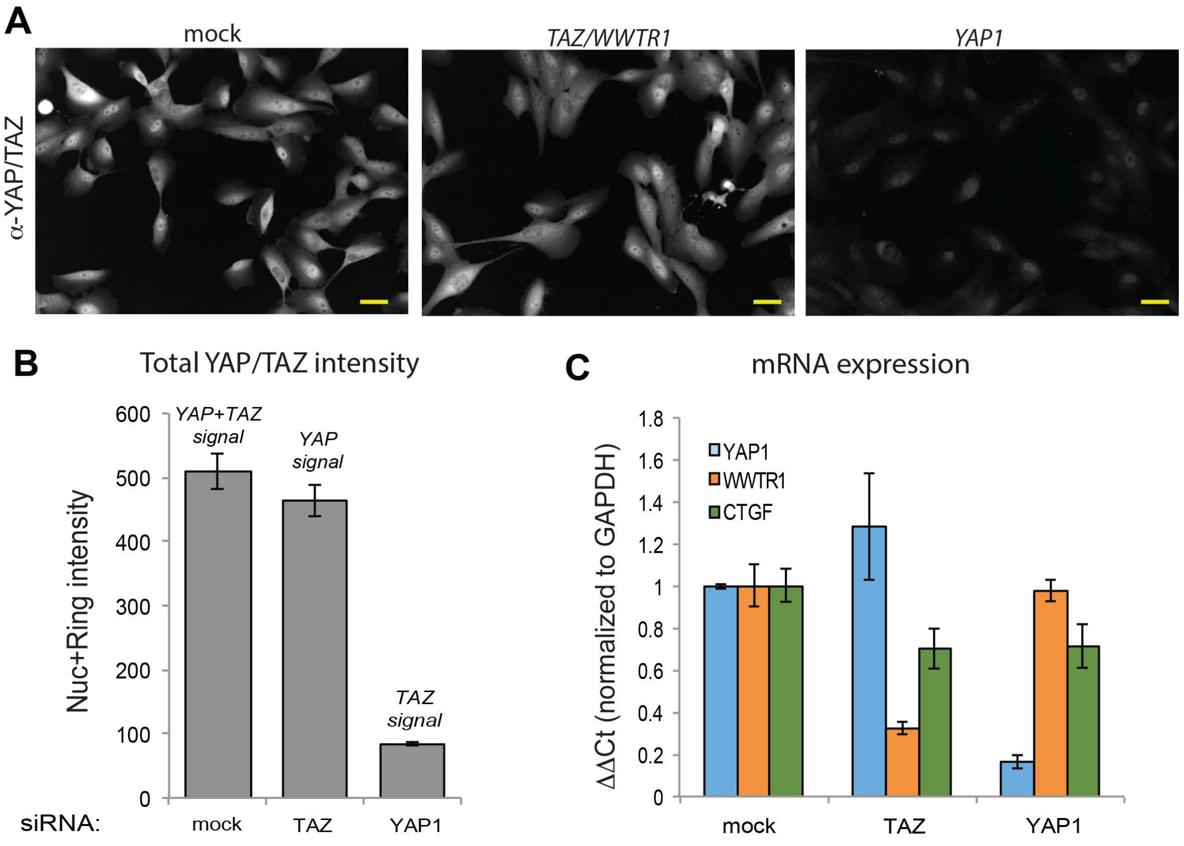

Figure S1. Related to Figure 1. YAP protein comprises the majority of immunofluorescence signal in MCF10A cells labeled with anti-YAP/TAZ (Santa Cruz 63.7) antibody. A) Mock- or siRNA-transfected cells (indicated) labeled with anti-YAP/TAZ antibody, identical exposures and settings. Scale bar = 20 μm. B) Quantification of total (nuclear + perinuclear) YAP/TAZ intensities. Mean +/- SD (*n* = 8 wells/condition). C) Confirmation of gene knockdown by qRT-PCR, normalized to *GAPDH* mRNA and control (wild type) (ΔΔCt).

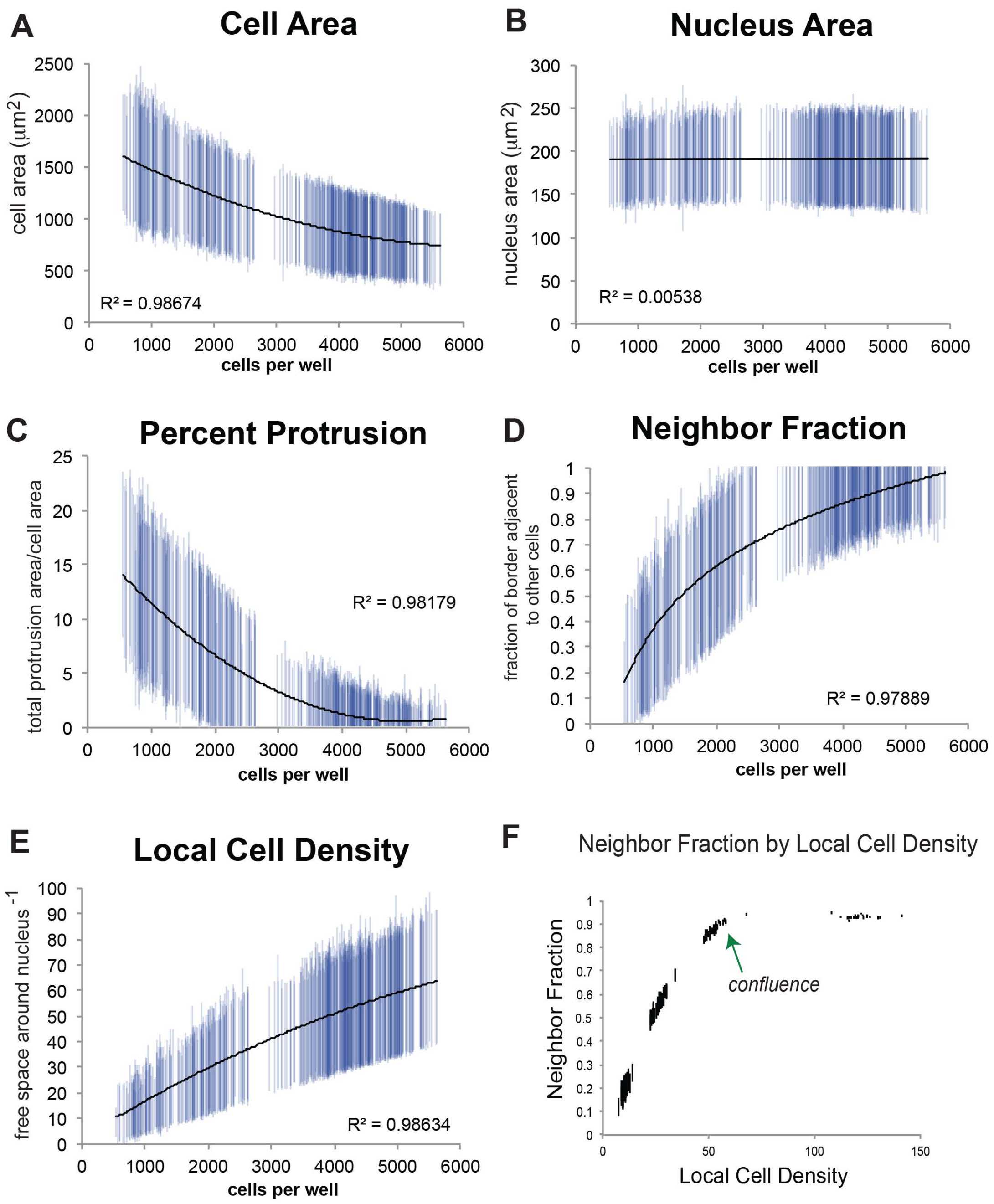

Figure S2. Related to Figure 2 and Supplementary Information 2. Normalization curves for density-dependent features. A-E) Mean +/- SD for all control wells from 10 screen plates (*n* = 554 wells, 273-3382 cells/well). Pearson's correlation coefficients (R^2) for average feature values and number of cells per well are indicated. Density-sensitive features for all samples were normalized using best-fit regressions of control wells (linear or non-linear), as shown in Supplementary Information 2. Uncorrelated features, such as nuclear area, were normalized to control well averages. F) Neighbor fraction by local cell density in MCF10A cells seeded at various initial numbers of cells/well. Confluence corresponds to LCD ~ 60. Mean +/- 99% CI per well.

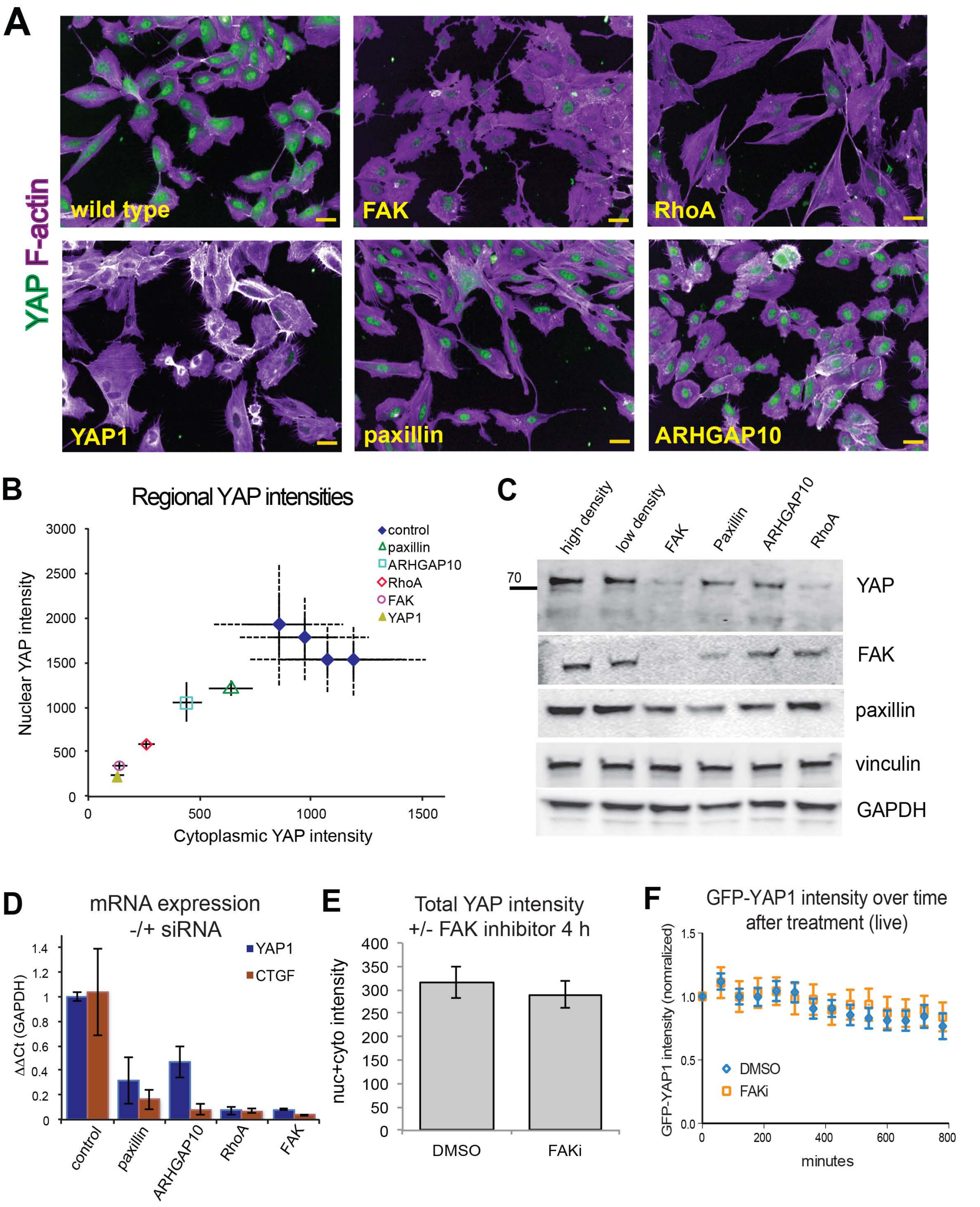

Figure S3. Related to Figure 2. Depletion of FAK and FA-associated genes leads to down-regulation of YAP. A) MCF10A cells transfected with the siRNA targeting the indicated genes. Green: anti-YAP (Santa Cruz 63.7). Purple: F-actin (phalloidin). B) Nuclear and cytoplasmic (perinuclear) YAP intensities in wild type and siRNA-transfected cells. Mean +/- SD (solid lines) and +/- 2 SD (dotted lines) for control wells. C) Western blots of cell plated at high or low density, or transfected with siRNA targeting the indicated genes. D) Expression of *YAP1* and *CTGF* mRNA measured by qRT-PCR, normalized to *GAPDH* mRNA and control (wild type) ΔΔCt. E) Total YAP intensity (nuclear+perinuclear) in MCF10A cells treated with DMSO or FAK inhibitor (2 μm PF-573288) for 4 h. F) Quantification of normalized GFP-YAP1 intensities in live cells. Mean +/- 95% CI (n = 12 cells/condition).

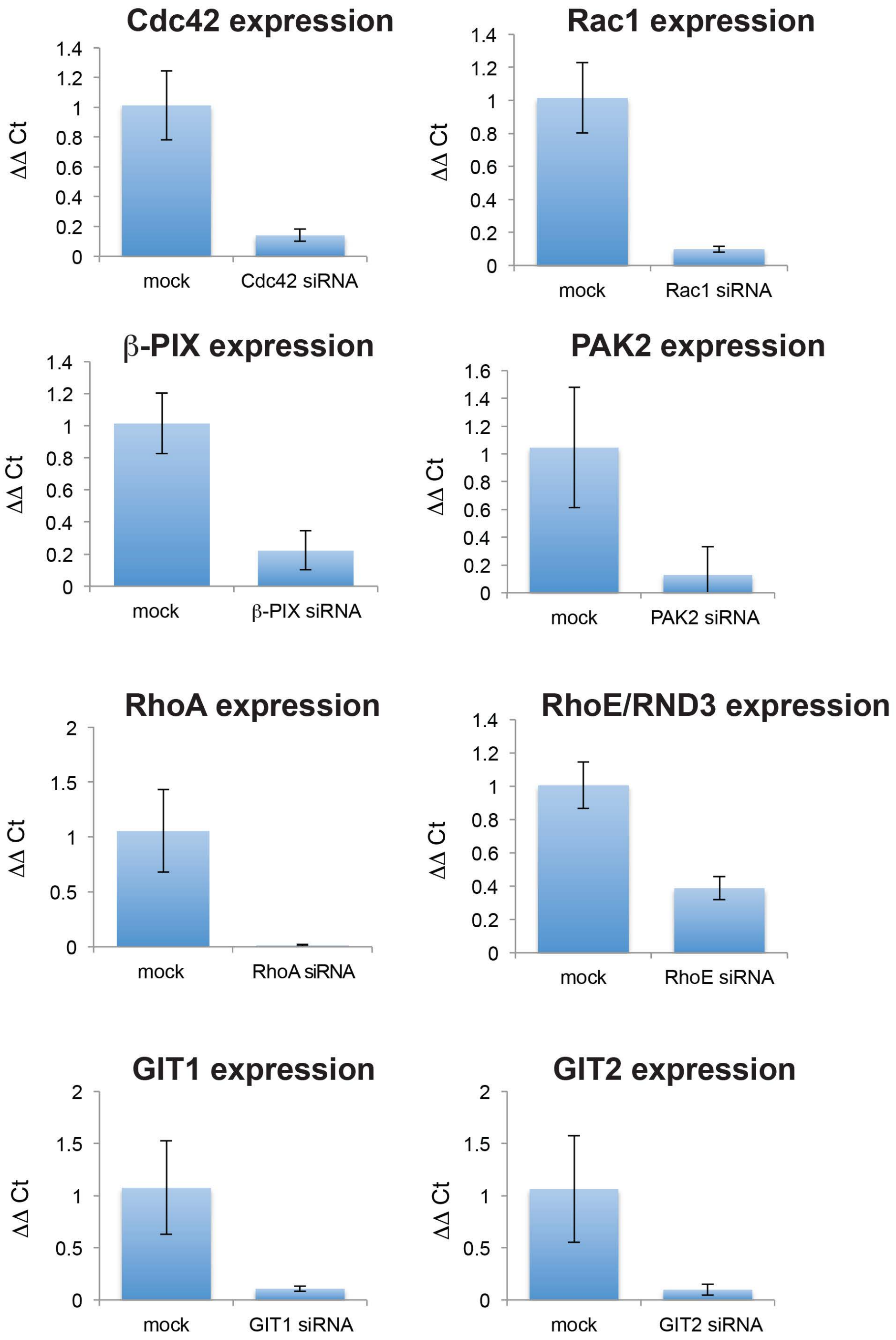

Figure S4. Related to Figure 3. Quantitative RT-PCR analysis of target gene mRNA levels normalized to GAPDH or β-actin in cells transfected for 72 h with the indicated siRNA (OnTarget Plus) or no siRNA (mock).

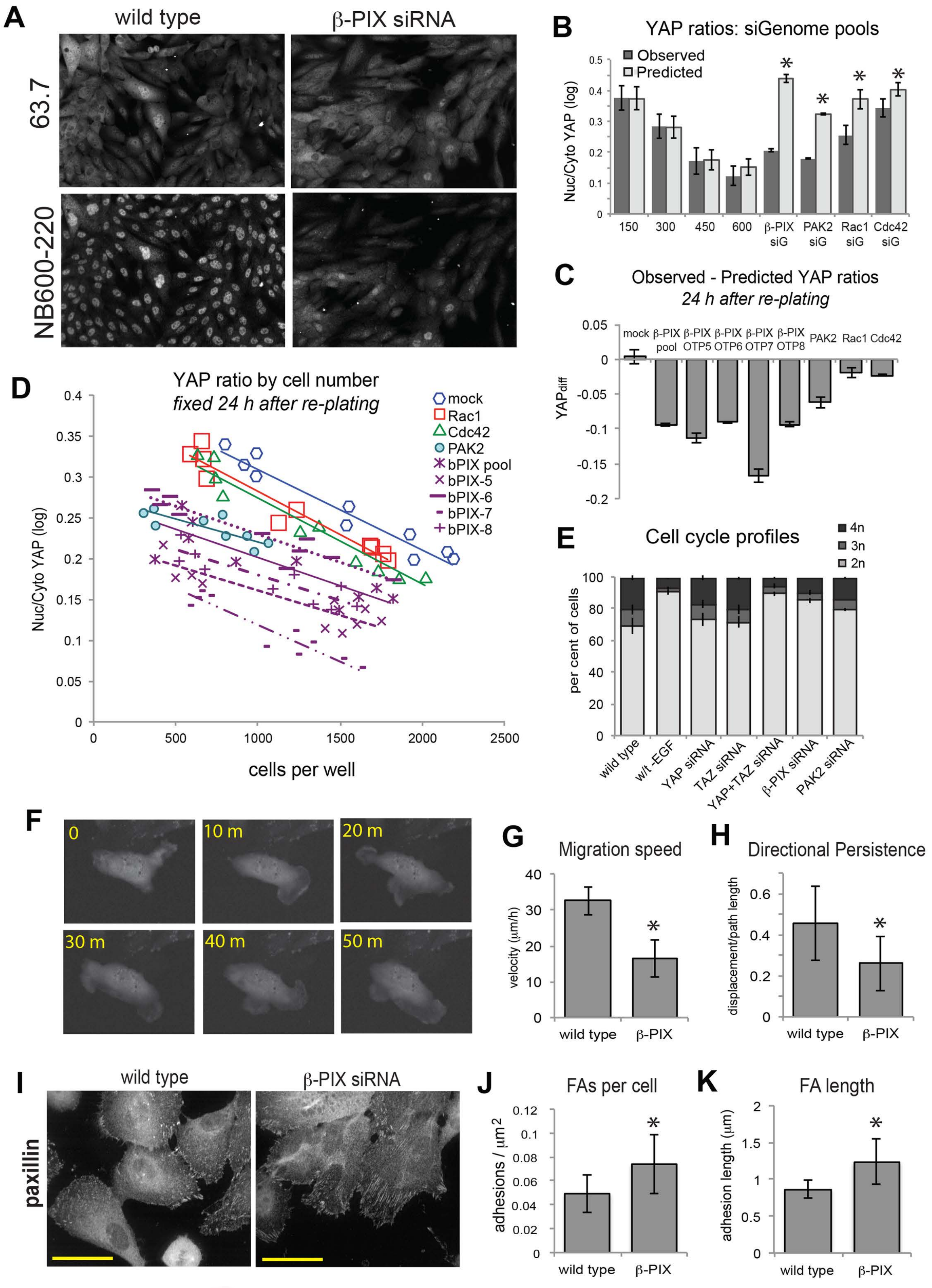
A
wild type
β-PIX siRNA
63.7
NB600-220
B
YAP ratios: siGenome pools
Observed
Predicted
Nuc/Cyto YAP (log)
150
300
450
600
β-PIX siG
PAK2 siG
Rac1 siG
Cdc42 siG
C
Observed - Predicted YAP ratios
24 h after re-plating
mock
β-PIX pool
β-PIX OTP5
β-PIX OTP6
β-PIX OTP7
β-PIX OTP8
PAK2
Rac1
Cdc42
YAPdiff
D
YAP ratio by cell number
fixed 24 h after re-plating
mock
Rac1
Cdc42
PAK2
bPIX pool
bPIX-5
bPIX-6
bPIX-7
bPIX-8
Nuc/Cyto YAP (log)
cells per well
E
Cell cycle profiles
4n
3n
2n
per cent of cells
wild type
w/t-EGF
YAP siRNA
TAZ siRNA
YAP+TAZ siRNA
β-PIX siRNA
PAK2 siRNA
F
0
10 m
20 m
30 m
40 m
50 m
G
Migration speed
velocity (μm/h)
wild type
β-PIX
H
Directional Persistence
displacement/path length
wild type
β-PIX
I
wild type
β-PIX siRNA
paxillin
J
FAs per cell
adhesions / μm2
wild type
β-PIX
K
FA length
adhesion length (μm)
wild type
β-PIX

FIGURE S5

Figure S5. Related to Figure 3. Validation and characterization of putative YAP regulators. A) Wild type and β-PIX knockdown cells labeled with two anti-YAP/TAZ antibodies targeting different domains. 63.7: unknown epitope. NB600-220: C-terminal PDZ-binding motif. B) Observed and predicted nuclear/cytoplasmic YAP ratios for siGenome siRNA pools. * $YAP_{diff} < 0.06$ (2 SD of controls). C) YAP_{diff} for sparsely plated cells transfected with the indicated siRNAs (48 h), trypsinized and re-plated for 24 h. Local cell densities (LCD) between 15 and 25 (confluence ~ 65; see Fig. S6D). D) Nuclear/cytoplasmic YAP ratios in wild type and knockdown cells re-plated at varying densities. E) Cell cycle profiles determined by FACS analysis of DNA content (2n-4n) 72 after siRNA or mock transfection. Wild type cells were also cultured in EGF-free medium for comparison. Mean +/- SD of biological replicates (n = 3). F) Time-lapse series of β-PIX-depleted cell labeled with CellTracker at 10 min intervals. G) Speed of randomly migrating wild type and β-PIX knockdown cells. * $p < 0.01$. H) Directional persistence (displacement/total path length) of randomly migrating wild type and β-PIX knockdown cells. * $p < 0.01$. I) Cell-ECM adhesions labeled by paxillin staining. Scale bar = 20 μm. J) Number of FAs /μm^2 per cell. K) Average FA length. Mean +/- SD (n = 50 cells).

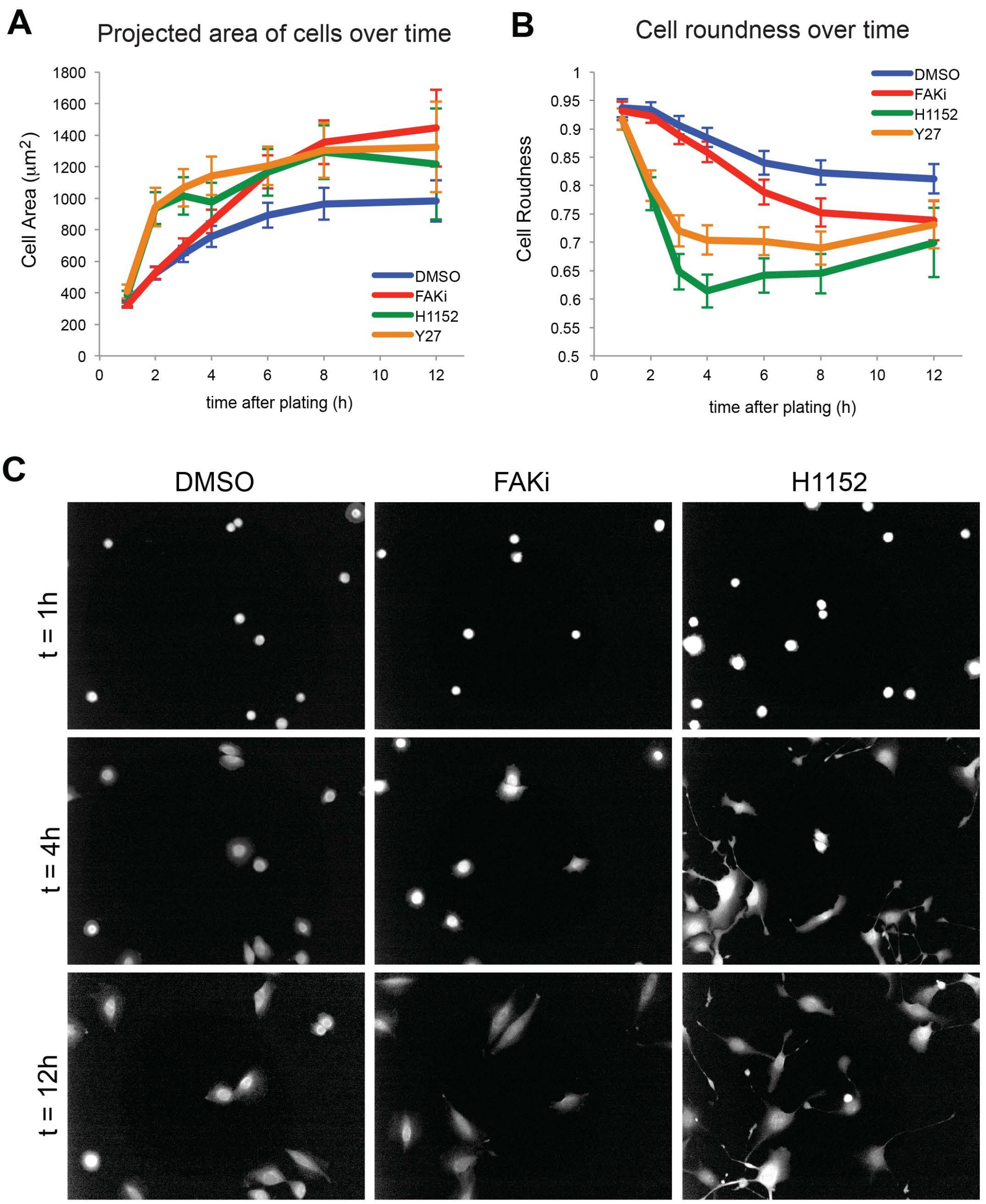

Figure S6. Related to Figures 4 and S9. Morphological analysis of spreading cells. Cells were labeled with CellTracker Orange (Invitrogen) prior to trypsinization, then seeded on fibronectin-coated plastic in medium containing DMSO (control), PF573288 (FAKi), H1152, or Y-27632 (Y27) and imaged every 5 min with a 20X water immersion lens (NA = 0.6) on the Opera Cell::Explorer microscope. Cell area and cell roundness (form factor) were measured following automated segmentation using Columbus (PerkinElmer). (A) Cell area +/- 95% CI. (B) Cell roundness +/- 95% CI. n = 45-100 cells/time point. (C) Representative images from indicated time points. Scale bars = 20 μm.

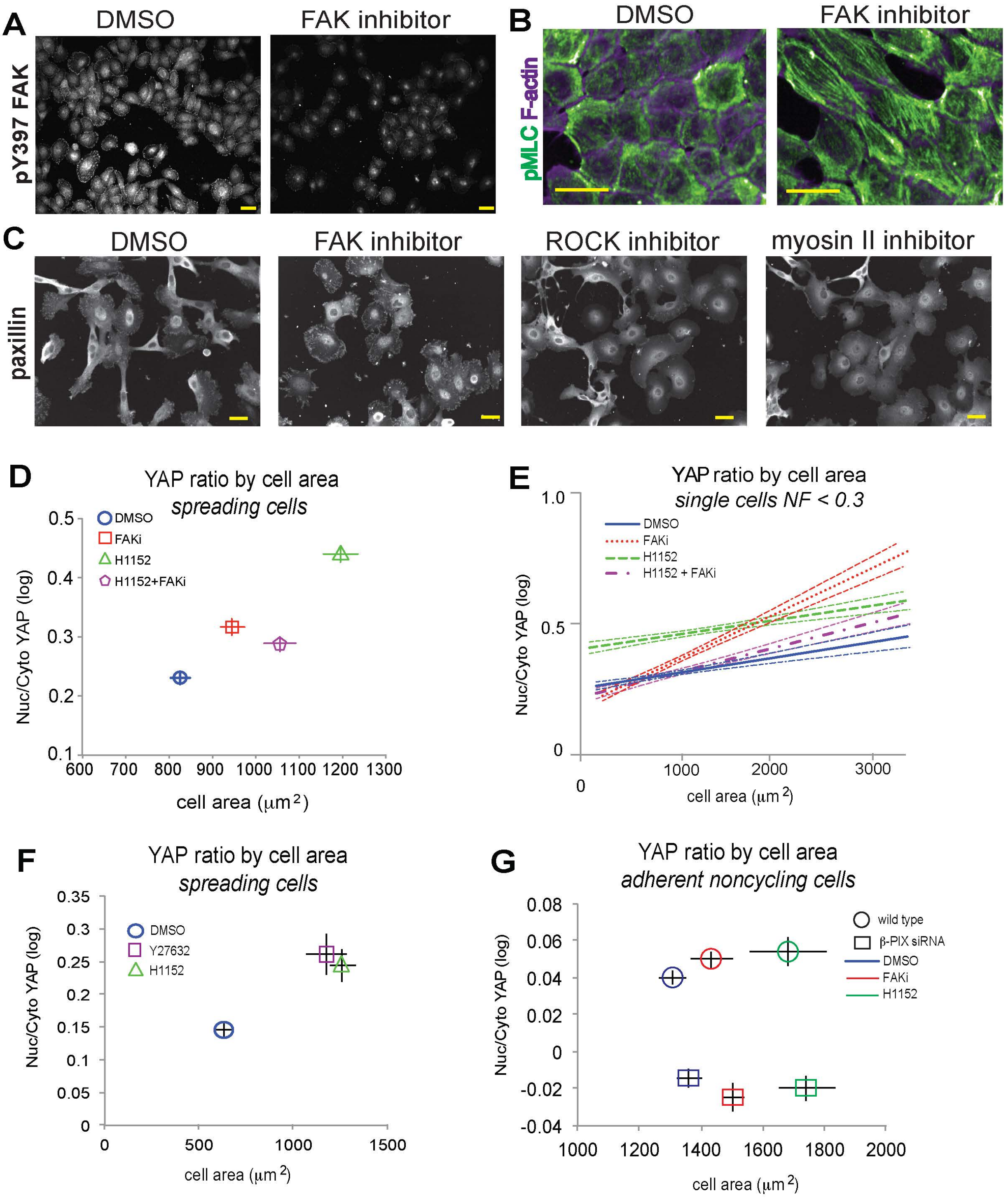
A
DMSO
FAK inhibitor
pY397 FAK
B
DMSO
FAK inhibitor
pMLC F-actin
C
DMSO
FAK inhibitor
ROCK inhibitor
myosin II inhibitor
paxillin
D
YAP ratio by cell area
spreading cells
DMSO
FAKi
H1152
H1152+FAKi
Nuc/Cyto YAP (log)
cell area (μm2)
E
YAP ratio by cell area
single cells NF < 0.3
DMSO
FAKi
H1152
H1152 + FAKi
Nuc/Cyto YAP (log)
cell area (μm2)
F
YAP ratio by cell area
spreading cells
DMSO
Y27632
H1152
Nuc/Cyto YAP (log)
cell area (μm2)
G
YAP ratio by cell area
adherent noncycling cells
wild type
β-PIX siRNA
DMSO
FAKi
H1152
Nuc/Cyto YAP (log)
cell area (μm2)

FIGURE S7

Figure S7. Related to Figure 4. Effect of FAK and ROCK inhibition on actomyosin, FAs, and YAP. A) Phospho-Y397 immunostaining of MCF10A cells treated with DMSO or FAK inhibitor (2 h). Exposure times, laser power, and all image contrast and brightness settings were identical. B) Cells treated with DMSO or FAKi stained for phospho-myosin II light chain (pMLC) (green) and F-actin (purple). Scale bar = 20 μm. C) Paxillin stained cells plated for 3 h on FN with the indicated inhibitors or DMSO (control). Scale bar = 20 μm. D) Nuclear/cytoplasmic YAP ratios by cell area for cells plated on fibronectin (FN) for 4 h in complete medium plus DMSO, FAK inhibitor (PF-573288) and/or ROCK inhibitor (H1152). Mean +/- SD (*n* = 4 replicate wells/condition). E) YAP ratio as a function of cell area for single cells with few cell-cell contacts (NF < 0.3) plated on FN. Lines indicate mean +/- 99% CI (*n* = 1500 cells/condition). F) YAP ratio by cell area in cells plated with DMSO, H1152, or Y-27632. Mean +/- SD (*n* = 4 replicate wells/condition). G) Wild type and β-PIX depleted cells plated for 72 h and EGF-starved for 48 h to synchronize in G0/G1 treated with DMSO, FAK inhibitor, or H1152 for 4 h. Mean +/- SD (*n* = 4 replicate wells/condition).

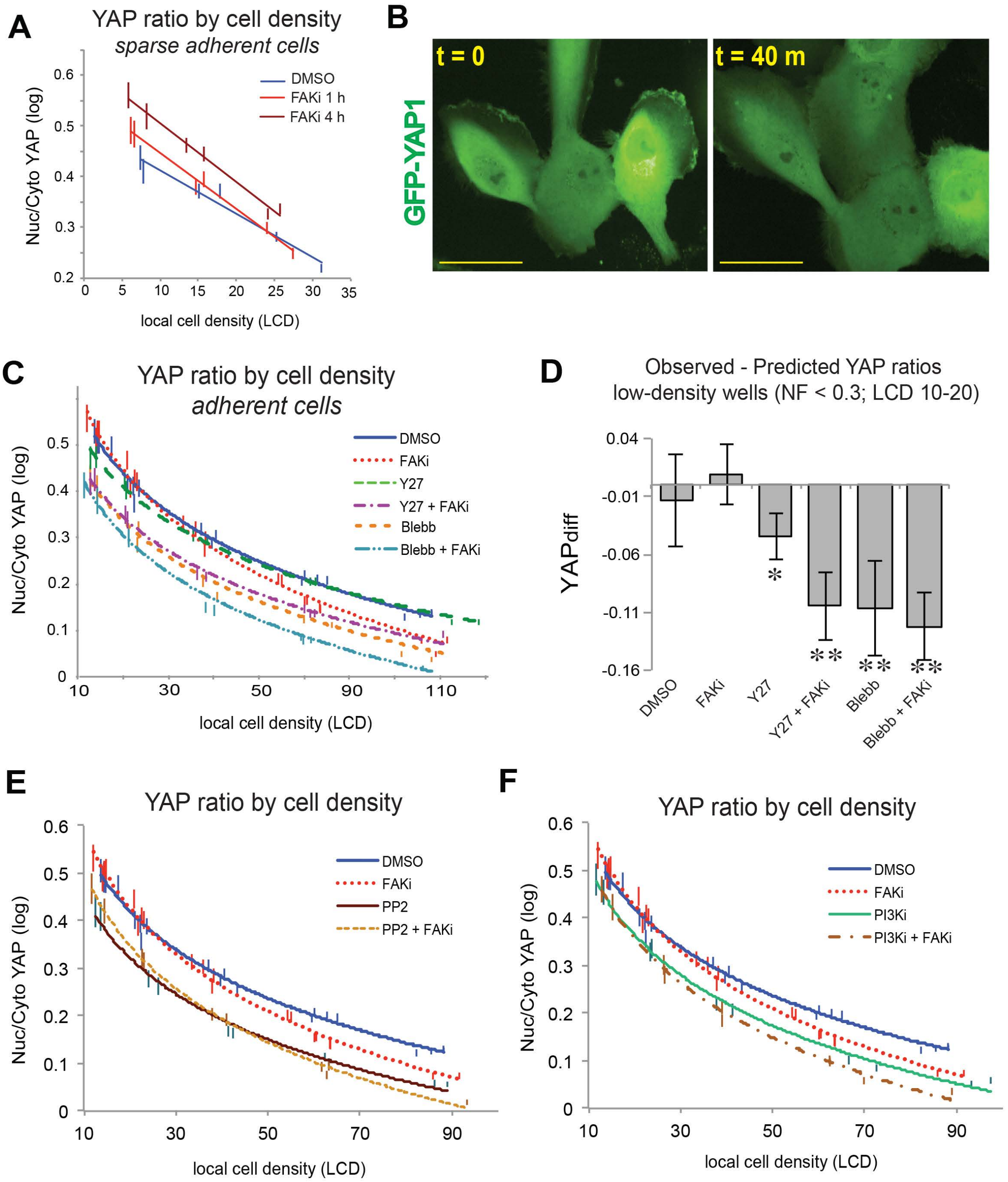
A
YAP ratio by cell density
sparse adherent cells
DMSO
FAKi 1 h
FAKi 4 h
Nuc/Cyto YAP (log)
local cell density (LCD)
B
t = 0
t = 40 m
GFP-YAP1
C
YAP ratio by cell density
adherent cells
DMSO
FAKi
Y27
Y27 + FAKi
Blebb
Blebb + FAKi
Nuc/Cyto YAP (log)
local cell density (LCD)
D
Observed - Predicted YAP ratios
low-density wells (NF < 0.3; LCD 10-20)
YAPdiff
DMSO
FAKi
Y27
Y27 + FAKi
Blebb
Blebb + FAKi
E
YAP ratio by cell density
DMSO
FAKi
PP2
PP2 + FAKi
Nuc/Cyto YAP (log)
local cell density (LCD)
F
YAP ratio by cell density
DMSO
FAKi
PI3Ki
PI3Ki + FAKi
Nuc/Cyto YAP (log)
local cell density (LCD)

FIGURE S8

Figure S8. Related to Figure 5. Effects of FAK, ROCK, myosin II, Src, and PI3K inhibition on YAP localization in adherent cells. A) Nuclear/cytoplasmic YAP ratio by local cell density in sparsely plated adherent cells treated with DMSO (4 h) or FAK inhibitor (1 h or 4 h). Mean +/- 99% CI per well. B) Live cells expressing GFP-YAP1 before and 40 min after addition of FAK inhibitor (2 μM PF-573288). Scale bar = 20 μm. C) Nuclear/cytoplasmic YAP ratio by local cell density. Mean +/- 99% CI per well with best-fit regression lines (logarithmic). D) YAP_{diff} for cells seeded at low densities treated with the indicated small molecule inhibitors or DMSO. Mean +/- SD for replicate wells (*n* = 4 wells/condition, 500-1200 cells/well). E-F) Nuclear/cytoplasmic YAP ratio by local cell density (LCD). Mean +/- 99% CI per well with best-fit regression lines (logarithmic). Src inhibitor = 10 μm PP2. PI3K inhibitor = 50 μm LY294002.

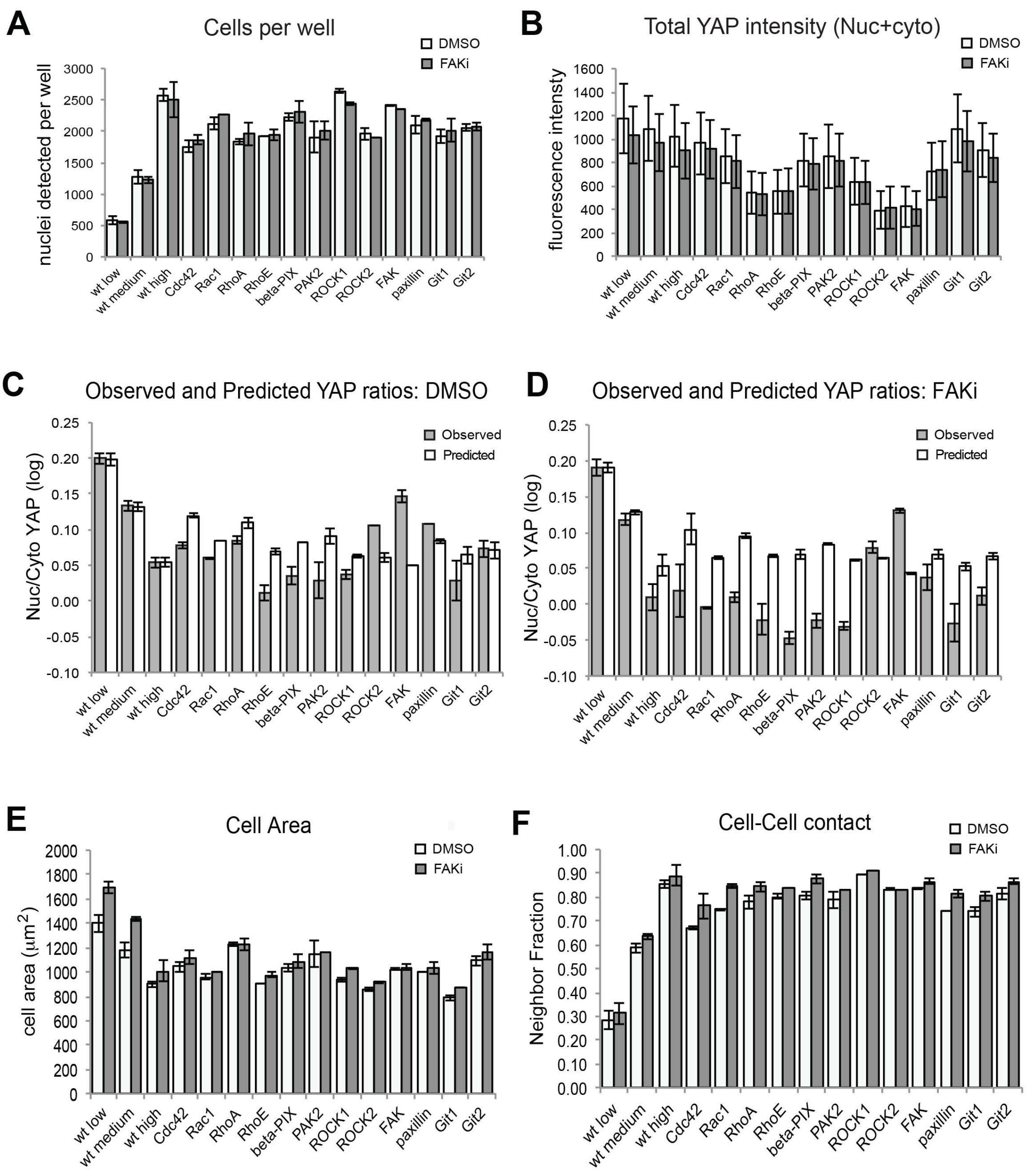

Figure S9. Related to Figure 5K. Characterization of MCF10A cells plated at low, medium, or high densities or transfected with siRNA for 48 h and treated with DMSO or FAK inhibitor (1 h). A) Nuclei detected per well. Mean +/- SD of replicate wells (*n* = 4 wells/condition). B) Total YAP intensity (nucleus + perinuclear ring region). Mean +/- average of SDs per well. C-D) Observed and predicted nuclear/cytoplasmic YAP ratios for DMSO- and FAKi-treated cells. E-F) Average cell area and neighbor fraction. Mean +/- SD of replicate wells.

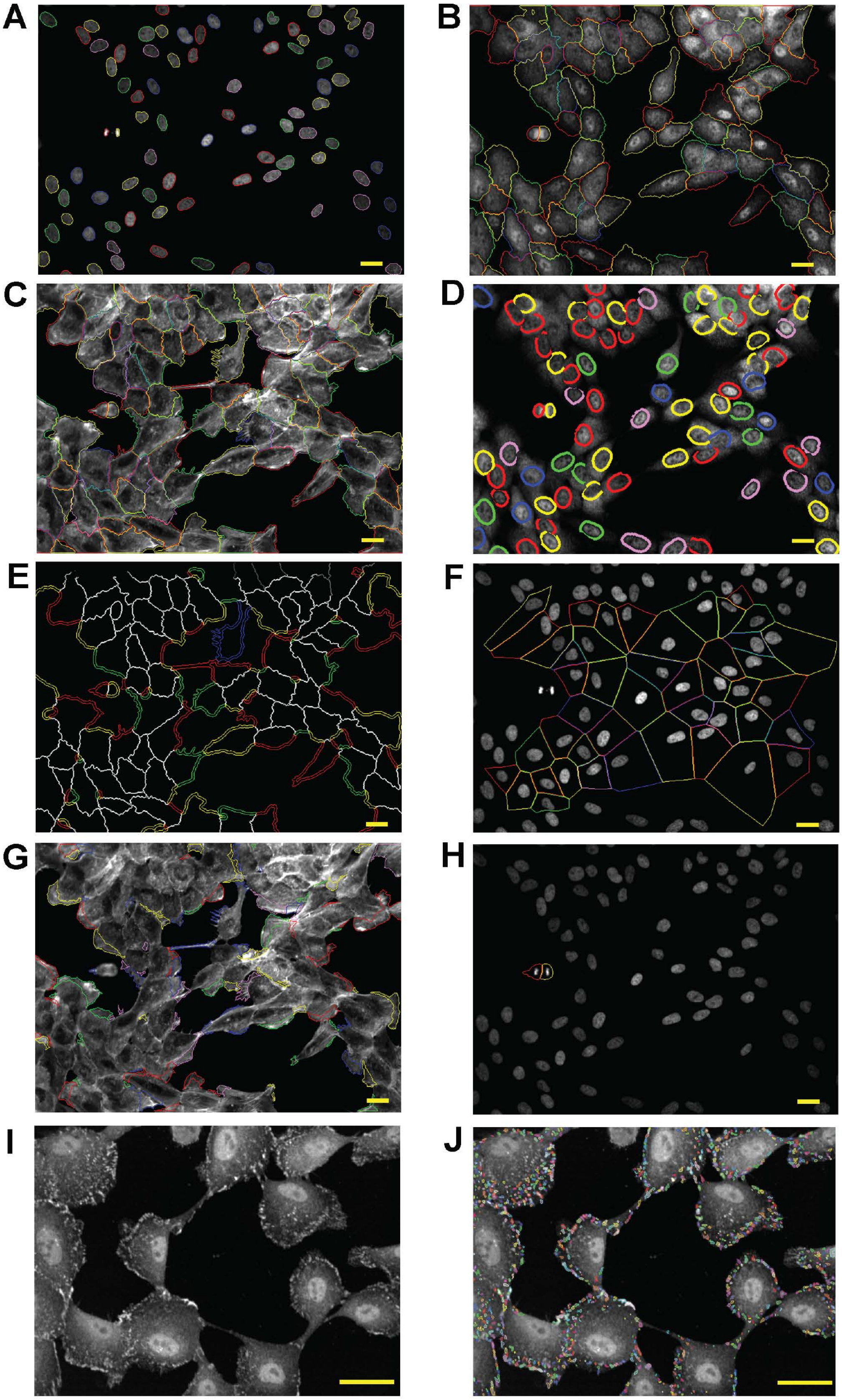

FIGURE S10

Figure S10. Related to Figure 1 and STAR Methods. Automated segmentation for feature extraction. A) Nuclei segmented on Hoechst (DNA) channel. B) Cell cores segmented on YAP channel using nuclei as seeds. C) Second segmentation step to identify cell bodies on actin channel using cell cores as seeds. D) Perinuclear ring region. E) Cell boundaries were divided into free edges (colored) and cell-cell borders (white) to determine Neighbor Fraction (NF). F) Local Cell Density was determined using Voronoi segmentation of nuclei. Cells whose borders were within 30 pixels of the edge of the field were not analyzed. G) Segmentation of protrusions. H) Mitotic cells were classified using multidimensional binning and excluded from analysis. I-J) Automated segmentation of focal adhesions using a spot finding algorithm. Scale bars = 20 μm.

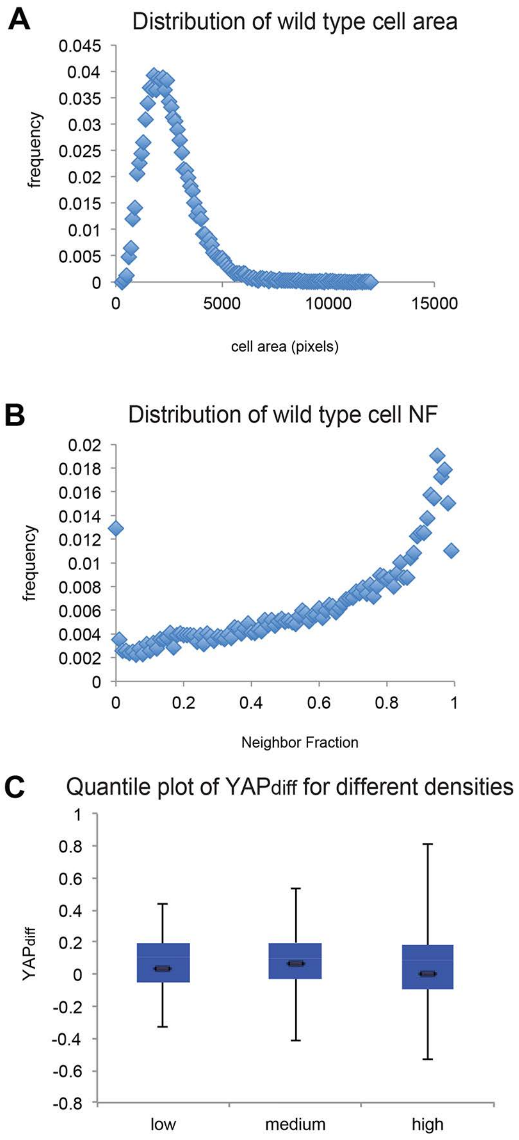

Figure S11. Related to Figure 2 and STAR Methods. Distributions of feature values and YAP_{diff} in wild type dataset. A) Frequency plot of cell area (pixels; 1 pixel = 0.64 μm). B) Frequency plot of NF. n = 29549 cells. C) Quantile plot of YAP_{diff} for low, medium, and high density wells.

Table S1. Related to STAR Methods. Correlation matrix of shape features and YAP ratio for wild type single cells (n < 25,000).

	NF	LCD	CellA	NucA	Nuc/CellA	ProX	Pro/CellA	ProN	ProA	YAP ratio
Neighbor Fraction (NF)	1	0.698	-0.498	0.016	0.566	-0.706	-0.66	-0.569	-0.59	-0.596
Local Cell Density (LCD)		1	-0.663	-0.226	0.676	-0.608	-0.524	-0.536	-0.481	-0.578
Cell Area (CellA)			1	0.459	-0.694	0.601	0.559	0.622	0.697	0.342
Nucleus Area (NucA)				1	0.045	-0.019	-0.054	0.071	0.066	-0.197
Nucleus area / cell area (Nuc/CellA)					1	-0.544	-0.507	-0.534	-0.47	-0.467
Protrusion Extent (ProX)						1	0.86	0.79	0.787	0.437
Total protrusion area / cell area (Pro/CellA)							1	0.688	0.906	0.339
Number of Protrusions (ProN)								1	0.634	0.347
Protrusion Area (ProA)								26039	1	0.3
YAP ratio										1

Table S2: Related to STAR Methods. PCR primers for qRT-PCR.

**Gene**	**Forward (5'-3')**	**Reverse (5'-3')**
ARHGEF7	CGAGAAAGTCTACCCCGAGC	GCCCGATGTCTGCTGTTACT
Cdc42	CGACCGCTGAGTTATCCACA	TTGACAGCCTTCAGGTCACG
CTGF	GAAGCAGAGCCGCCTGTGCA	ACCGGCAGGGTGGTGGTTCT
FAK/PTK2	AGTAAAATCCAGCCAGCCCC	GACATACTGCTGGGCCAGTT
Git1	GATGTTAATGGCCGCACACC	TGAGACATGCACTTTTGCCG
Git2/Pkl	CAAACCGGCAGAAGAGCCTA	GCTGCTGTATCTTGGCCTCA
PAK2	TCGCCATTGCCGAAGG	GAGGTGCTGGAGGCTTATCT
PXN	GGAAAAGTTGCGGGGCATAG	CAAGAACACAGGCCGTTTGG
Rac1	GGGAGACGGAGCTGTAGGTAA	AGAACACATCTGTTTGCGGA
RhoA	ATCCCAGAAAAGTGGACCCC	GCCTTCTTCAGGTTTCACCG
RhoE	GGTGGGAGACAGTCAGTGTG	GAAGTGTCCCACAGGCTCAA
ROCK1	GAGCAGAAGTGCAGAACCTCAA	ACAGCTGTGTCCGATTCTGT
ROCK2	GCCGCCGTTGCCATATTAAG	GGCAGTTAGCTAGGTTTGTTTGG
WWTR1/TAZ	TATGGGACAGTCCGGGAGC	CGAGGCTTGGCTGACAAATC
YAP1	AACAGCAAGAACTGCTTCGG	TTTGAGTCCCACCATCCTGC
GAPDH	AGATCCCTCCAAAATCAAG	GGCAGAGATGATGACCCTT
